# Adhesive and Hemostatic Hydrogel for the Management of Postpartum Hemorrhage

**DOI:** 10.1021/acsami.6c05034

**Published:** 2026-06-22

**Authors:** Sarah E. Miller, Prasenjeet Ingole, Anya Stolyarova, Dylan Po, Tripp D. Moss, Jasmine Sureka, Saptarshi Biswas, Ryan Davis, Akhilesh K. Gaharwar

**Affiliations:** † Department of Biomedical Engineering, College of Engineering, 14736 Texas A&M University, College Station, Texas 77843, United States; ‡ Department of Biology, College of Arts and Sciences, 14736 Texas A&M University, College Station, Texas 77843, United States; § School of Public Health, 14736 Texas A&M University, College Station, Texas 77843, United States; ∥ Department of Materials Science and Engineering, College of Engineering, Texas A&M University, College Station, Texas 77843, United States; ⊥ Interdisciplinary Program in Genetics, Texas A&M University, College Station, Texas 77843, United States; # Center for Remote Health Technologies and Systems, Texas A&M University, College Station, Texas 77843, United States

**Keywords:** women’s health, adhesive, hemostatic, injectable, hydrogel, postpartum hemorrhage

## Abstract

Postpartum hemorrhage is the number one cause of maternal mortality across the globe due to rapid and excessive blood loss. This condition frequently results from uterine atony, wherein the uterus is unable to contract itself against bleeding uterine vessels. As a result, additional management techniques are often required in order to prevent this condition from progressing to death. In this study, we present a hydrogel with hemostatic and wet tissue adhesion properties as a potential treatment for postpartum hemorrhage. This hydrogel demonstrates improved adhesivity compared to controls in lap shear and burst strength evaluations. In addition, this material demonstrates hand injectability at speeds up to 5 mL/min, good hemostatic ability in vitro, and short-term cyto- and hemocompatibility. We have evaluated this hydrogel in a rat model of uncontrolled hemorrhage and in a custom benchtop model of the postpartum uterus to contextualize these findings and to grant an understanding of this material in physiological settings. Finally, we consider potential routes of degradation and removal of the biomaterial from the uterus following successful hemorrhage control. Overall, the current hemostatic hydrogel integrates tissue adhesion, rapid hand injectability, reduced clotting time, and short-term biocompatibility, demonstrating effective bleeding control in benchtop and rat hemorrhage models.

## Graphical abstract

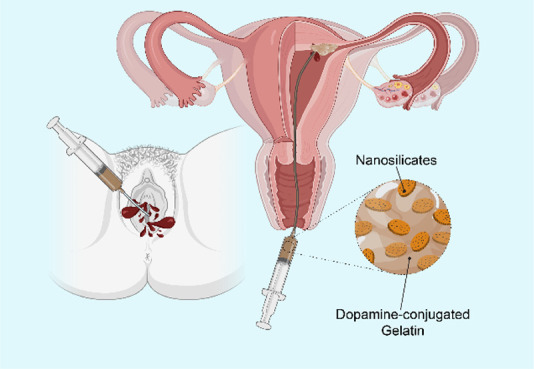

## Introduction

Despite improvements in obstetrics, postpartum hemorrhage (PPH) remains the worldwide leading cause of maternal mortality^1^ and has become increasingly prevalent over the last 20–30 years.^2^ PPH is defined as blood loss greater than 1000 mL after childbirth with signs of hypovolemia. The vast majority PPH occurs within 24 h of delivery^3^ and is most often attributed to uterine atony,^1^ which presents with a deficit of myometrium contractions that are typically responsible for stopping uterine hemorrhage. This bleeding is difficult to localize and is noncompressible, posing considerable challenges for efficient management. Other contributing factors to PPH include retained placental tissue, genital tract lacerations, uterine inversion, and coagulation disorders, each of which can present additional challenges.^2,3^ In addition to the risk of death, PPH poses a number of health risks, including anemia, renal failure, pituitary gland damage known as Sheehan’s Syndrome, and psychological disorders.^4^ Furthermore, PPH often presents in patients without any known risk factors,^2,4,5^ so immediate access to reliable emergency obstetric and hemostatic care is essential to patient survival.

Current treatments for PPH include a wide range of drugs, manual maneuvers, and surgical strategies.^2^ These treatments focus on correcting the underlying cause, which can be categorized into four main groups: tone (uterine atony), tissue (retained placental tissue), trauma (damage to the uterus and genital tract), or thrombin (coagulation disorders).^5–8^ Oxytocin is considered the gold standard, first-line defense against PPH.^1^ This hormone, whether naturally occurring or synthetic, continues generating strong contractions in the uterus following delivery, enabling the uterus to compress the bleeding vessels present in its tissue. Oxytocin has demonstrated success when given prophylactically;^5^ however, prolonged exposure can lead to uterine receptor desensitization, reduced effectiveness, and continued hemorrhage.^9,10^ There is some evidence that the rise in PPH may be linked to a rise in uterine atony incidence, which in turn may be attributed to higher rates of induction of labor and prophylactic oxytocin administration.^2,6^ This has prompted research into alternative uterotonic medications, such as oxytocin analogues, prostaglandins, and ergot alkaloids^5^ along with nonmedication-based therapies.^11^

Manual or physical treatments include massaging the uterus, repositioning an inverted uterus, or applying an intrauterine balloon to tamponade the bleeding and can escalate to more invasive and technically challenging actions such as arterial embolization, massive transfusion protocols, or hysterectomy.^2,8,12^ Each of these procedures required skilled providers and can increase the risk of further damage to the patient’s tissues, necrosis, thromboembolic events,^2^ or limitations to future fertility.^7^ Further, the use of blood transfusions has been correlated with a greater length of hospital stay, and these treatments often can be logistically challenging or cost-prohibitive.^2^ Based on these challenges, there continues to be a clinical need for novel treatments which can be applied by unskilled providers or in under-resourced areas while still maintaining high efficacy. Injectable biomaterials which can be applied locally onto the bleeding tissue offer an opportunity to address PPH directly while maintaining ease of use.

Hemostatic hydrogels have been used in numerous forms and for a myriad of applications, including peripheral vascular embolization,^13^ enterocutaneous fistulas,^14^ and as surgical adjuncts.^15,16^ Injectable biomaterials in particular offer advantages over traditional hemostatic materials in that they can conform to complex geometries and provide increased contact between the material and the tissue surface compared to gauzes or powders.^17^ These adhesive hydrogels can act as a sealant resulting from the polymeric composition and any functional group modifications present in the hydrogel,^17^ thus providing hemostatic action across the surface of a complex tissue geometry such as that present in the uterus. Additionally, injectable materials are often delivered in a minimally invasive manner, which can decrease the need for secondary trauma from surgical intervention.^17^ Current hemostatic materials in the form of powders, sponges, and mechanical tamponade devices such as balloons offer unique advantages for PPH, but are often limited to external applications or short-term use,^17^ requiring further management or invasive actions. Additionally, many current treatments for PPH require access to the uterus (e.g., compression sutures, manual compression, packing with gauzes, etc.),^2,8,12^ and often may not be immediately implemented in the case of cesarean sections or other disruptions to the uterine cavity.^18^ Injectable and adhesive hydrogels offer a potential solution to bridge the gap between current hemostatic materials and treatments and these clinical challenges.

Gelatin and its derivatives are a popular choice for fabricating such hydrogels due to low cost, strong biocompatibility, and ease of functionalization. Additionally, gelatin, as an ampholytic polymer with both positive and negative functional groups, has been utilized in hemostatic materials for its ability to form a mechanical barrier against bleeding. Dopamine and polydopamine, which can be conjugated to a gelatin backbone, are a popular choice for conferring wet-tissue adhesion to polymeric biomaterials as the numerous catechol groups present ready adhesion to biological tissues.^15^ The incorporation of wet-tissue adhesion is of particular interest for PPH management due to the high blood flow rates and volumes seen in the clinical presentation.

Herein, we present a multimodal hemostatic biomaterial composed of polydopamine, gelatin, and nanosilicates. This hydrogel aims to take a combinatorial approach to PPH management by utilizing both wet-tissue adhesion and hemostatic nanosilicates to address severe bleeding. We evaluate the wet-tissue adhesive ability and hemostatic ability to understand feasibility at managing uncontrolled bleeding. Further, we assess hemo- and cytocompatibility of the hydrogel in consideration of clinical use. Finally, we demonstrate successful management of uncontrolled hemorrhage in an in vivo rat model and contextualize the hydrogel with a custom benchtop model of the postpartum uterus as well as application to an in vivo rat uterus. Although this hydrogel exhibits some limitations in potential for standalone management of PPH, this material poses a viable alternative adjunct for improved management of PPH.

## Results and Discussion

### An Optimized Polydopamine–Gelatin and Nanosilicate Composite Hydrogel Demonstrated Improved Adhesion

In order to devise a hydrogel with wet-tissue adhesion functionality, we sought to incorporate polydopamine onto a gelatin polymer backbone (Figure 1A). Dopamine has been used widely in the design of adhesive biomaterials due to its multimodal adhesive action. The ring structure within the catechol present in dopamine is able to adhere to biological tissues through π–π stacking, while the oxygen atoms present in the catechol enable hydrogen bonding as well as covalent reactions to amines and thiols present in biological tissues via Michael addition and Schiff base reactions.^16,19^ We performed chemical conjugation of polydopamine to the gelatin polymer using 1-ethyl-3-(3-(dimethylamino)­propyl) carbodiimide (EDC) and *N*-Hydroxysuccinimide (NHS) chemistry based on previously published literature^20–25^ (Figure S1A). In order to produce a polymer with high adhesion, we evaluated multiple process parameters for this synthesis, including reagent ratios (Figure S1B), synthesis step timing (Figure S1C), buffer solution (Figure S1D), reaction pH (Figure S1E), and synthesis sequence (Figure S2A). Additional discussion on these adjustments can be found in the Supporting Information. The resulting polymers displayed a brown coloring, indicating that polydopamine had formed (PDA-gelatin), rather than remaining as unpolymerized dopamine following conjugation.

**Figure 1:**
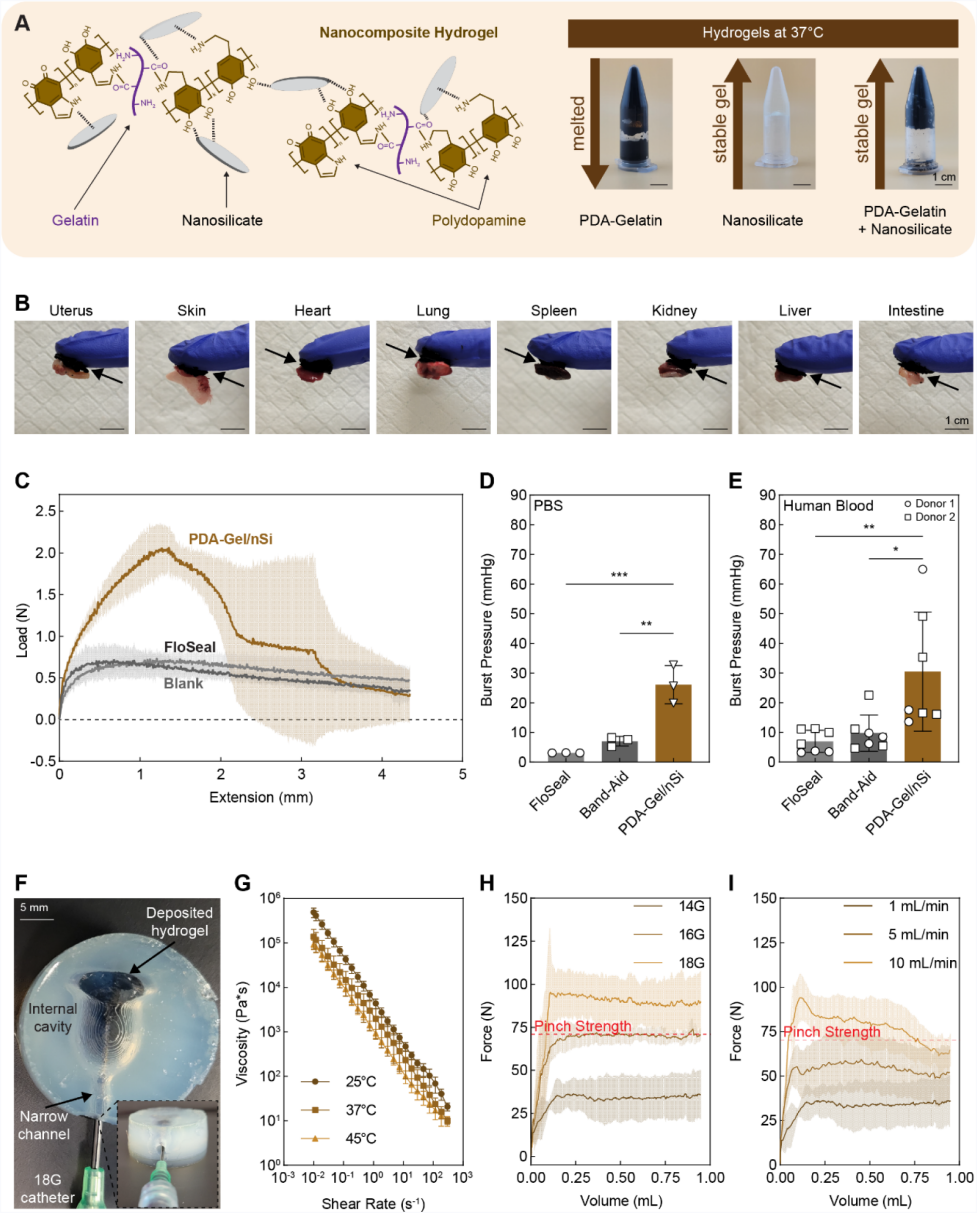
Fabrication and evaluation of adhesive, injectable hydrogel. A) Schematic of hydrogel synthesis and composition. Inset shows hydrogel components (PDA-gelatin, nanosilicate) and complete hydrogel at 37 °C. The PDA-gelatin component is observed to be melted, while the nanosilicate component and hydrogel display stable gelation. B) Adhesion of hydrogel to various ex vivo rat tissues is assessed visually. Tissues shown are uterus, skin, heart, lung, spleen, kidney, liver, and intestine. Black arrows point to hydrogel layer between tissue sample and nitrile glove. C) Lap shear testing based on ASTM F2255–24. Load vs extension is shown for blank samples, PDA-gel/nSi, and FloSeal. *N* = 4; data shown are mean ± standard deviation. D) Burst strength testing with PBS as pumped fluid. PDA-gelatin/nSi hydrogel is compared against commercial Band-Aid, and FloSeal. FloSeal serves as a clinical hemostat sealant control. *N* = 3; data shown are mean ± standard deviation. Ordinary one-way ANOVA with Tukey’s multiple comparisons test. *p < 0.05, **p < 0.01, ***p < 0.001, ****p < 0.0001. E) Burst strength testing with fresh, citrated whole human blood as pumped fluid. PDA-gelatin/nSi hydrogel is compared against commercial Band-Aid, and FloSeal. FloSeal serves as a clinical hemostat sealant control. *N* = 7 across 2 unique donors (*N* = 3–4 each donor); data shown are mean ± standard deviation. Ordinary one-way ANOVA with Tukey’s multiple comparisons test. *p < 0.05, **p < 0.01, ***p < 0.001, ****p < 0.0001. F) Mini uterus model showing injectability. The hydrogel is shown injecting through a narrow channel via an 18-gauge catheter into an open cavity. Inset shows the side view with the catheter entering a narrow channel. G) Shear rate sweep of PDA-gelatin/nSi hydrogel at 25 °C, 37 °C, and 45 °C. *N* = 3; data shown are mean ± standard deviation. H) Injection force for PDA-gelatin/nSi hydrogel with 14-, 16-, and 18-gauge blunt tip needles with a flow rate of 1 mL/min. Note that data from 14-gauge needle appear in both Figure 1H and Figure 1I. Data are truncated at 0.95 mL to exclude increases in force resulting from the syringe plunger impacting the end of the syringe barrel. *N* = 3; data shown are mean ± standard deviation. I) Injection force for PDA-gelatin/nSi hydrogel at 1, 5, and 10 mL/min injection rates using a 14-gauge blunt tip needle. Note that data from 14-gauge needle appear in both Figure 1H and Figure 1I. Data are truncated at 0.95 mL to exclude increases in force resulting from the syringe plunger impacting the end of the syringe barrel. *N* = 3; data shown are mean ± standard deviation.

We evaluated these synthesized polymers for their catechol content determined quantitatively via Arnow’s method^26^ (Figure S1B-E, Figure S2B) and to identify the process parameters to maximize this functionalization. Additionally, we confirmed the successful conjugation of polydopamine to the gelatin backbone using proton nuclear magnetic resonance spectroscopy (^1^H NMR) (Figure S2C) and Fourier transform infrared spectroscopy (FTIR) (Figure S2D). A characteristic peak for conjugated catechols appeared in the NMR spectra at 2.7 ppm indicating methylene groups close to phenyl groups,^14,20,24^ which was absent in the pure gelatin spectrum (Figure S2C). FTIR analysis revealed somewhat broader peaks in the 3000–3500 cm^–1^ range, indicating the presence of the catechol^14,20^ (Figure S2D). To select the final polymer composition and synthesis procedure, we evaluated the adhesive ability of various resulting polymers using a weight-based lap shear-style adhesion test (Figure S3). A sample was placed between two porcine skin sections, suspended midair, and the lower section was progressively weighted until it fell. The total mass of the lower skin section and the added weight was used to calculate the force on the sample and thus provide a rudimentary comparison of the adhesive ability for various materials. We selected a polymer synthesis involving presynthesis of polydopamine followed by conjugation to gelatin in an alkaline environment due to demonstrated improved adhesion over unmodified gelatin. Literature suggests that the incorporation method of dopamine appears to play a role in its adhesive ability: hydrogels containing unbound, unpolymerized dopamine displayed weakened adhesion, attributed to disruption of hydrogel chains,^27^ while materials containing polydopamine demonstrated a higher adhesive ability,^27^ suggesting that the increased catechol content in polydopamine can overcome the limited cohesion to improve adhesivity of a biomaterial. Additional discussion regarding the synthesis variations is available in the Supporting Information.

We noted that the PDA-gelatin polymer solutions were liquid and did not solidify at room temperature (Figure 1A), unlike unmodified gelatin which forms a solid construct at room temperature. This phenomenon is likely due to the presence of the conjugated polydopamine on the gelatin backbone, which is suspected to contribute steric hindrance among the gelatin chains along with consumption of carboxyl groups, thus preventing physical cross-linking and the formation of helical structures. This issue was mitigated upon combining the PDA-gelatin solution with an exfoliated nanosilicate (nSi) solution to result in a PDA-gelatin/nSi nanocomposite hydrogel (PDA-gel/nSi) (Figure 1A) that did not exhibit noticeable macroscale melting at physiological temperature. Nanosilicates, with their charged surfaces, can serve as multivalent ions capable of forming ionic cross-links with polymer chains.^28^ Thus, the inclusion of nanosilicates in the hydrogel design can enable this modified hydrogel to retain material cohesion and temperature stability. We performed a concentration sweep of PDA-gelatin and of hydrogels composed of PDA-gelatin and nanosilicates to assess hemostatic ability (Figure S4A-B) and adhesive ability (Figure S4C). Overall, a hydrogel composed of 10% PDA-gelatin and 10% nanosilicate demonstrated the balance of these two properties (Figure S4D). Therefore, we continued our study with this optimized hydrogel composition and evaluated this composite hydrogel for adhesivity, hemostatic ability, and potential for use as a treatment for postpartum hemorrhage.

We evaluated the optimized, PDA-conjugated hydrogel (10% PDA-gelatin 10% nanosilicate) for its adhesive ability in physiological conditions. We observed that the hydrogel was able to adhere to a variety of freshly harvested, ex vivo rat tissues, including the uterus and skin, and lift sections of these tissues against gravity (Figure 1B), indicating the potential for adhesion to biological tissues. Additionally, we conducted a lap shear test based on ASTM F2255–24, which determines the shear strength of tissue adhesives. In this test, two porcine skin sections approximately 3.0 cm × 1.5 cm × 2.5 mm are bonded in an overlapping fashion. The sample is allowed to bind the tissues for 30 s under 1–2 N of force, and subsequently loaded at a rate of 5 mm/min. In all samples, we observed that the two pieces of tissue initially resisted separation and then were slowly pulled apart (Figure 1C). We observed that the PDA-gel/nSi hydrogel demonstrated the highest maximum loading force (2.33 ± 0.31 N) over FloSeal (0.78 ± 0.11 N), and skin-to-skin samples (blank; 0.82 ± 0.19 N), representing a statistically significant increase (Figure S5A). This maximum load is converted to shear strength by dividing the maximum load by the bond surface area. PDA-gel/nSi exhibited a shear strength of 8830.9 ± 1252.0 Pa, which represented a significant improvement over FloSeal (3369.7 ± 297.5 Pa) and blank specimens (3713.9 ± 951.9 Pa) (Figure S5B). This adhesive strength is comparable to other dopamine-based bioadhesives previously reported at 10.20 ± 3.11 kPa^15^ and at 7.9 ± 1.8 kPa.^29^ Conversion of this shear strength to mmHg (Figure S5C), which identifies the PDA-gel/nSi hydrogel’s shear strength at 66.2 ± 9.4 mmHg, can yield a more thorough understanding of the adhesive ability in conjunction with burst strength testing, described below.

Following testing, we observed hydrogel on both pieces of porcine skin, suggesting that the mode of failure of the PDA-gel/nSi hydrogel is cohesive failure and that modulation of hydrogel cross-links may yield a higher performance material. In addition, we observed that the PDA-gel/nSi hydrogel displayed the longest time to maximum load (Figure S5D) at 20.58 ± 9.80 s. While this is not significantly higher than either FloSeal or blank samples, the numerical increase may suggest that the stronger cross-linking network in the hydrogel can improve adhesive ability. In addition to lap shear testing, burst strength testing provides a mechanism for evaluating continued adhesion under dynamic, pressurized flow conditions. Burst strength testing was conducted based on ASTM F2392–24, which determines the burst strength of surgical sealants.^30^ A section of porcine skin was perforated with a biopsy punch and tubing was secured inside this defect. The tubing was connected to a syringe and a syringe pump which pumped fluid at a constant rate of 2 mL/min.^30^ The sample was applied over the defect and the maximum pressure generated while pumping the fluid was determined. Burst strength testing revealed a significant improvement in the PDA-gelatin/nSi hydrogel compared to FloSeal in the presence of either phosphate-buffered saline (PBS) (Figure 1D) or citrated human blood (Figure 1E).

It is important to note that the results presented by the lap shear and burst strength tests indicate that the PDA-gel/nSi hydrogel would be unable to withstand the pressures present in PPH (roughly 70–80 mmHg in the spiral arteries^31,32^ ) when applied as a singular treatment. However, in both tests, the results do indicate an improvement compared to clinical product FloSeal, a commercial hemostatic agent composed of thrombin and gelatin granules, which has been used as a hemostatic sealant in cases of PPH.^33–35^ FloSeal is in a liquid state upon application which limits its ability to resist pressure and easily flows away from the site of application. Of note, reports describe the use of FloSeal in conjunction with applied pressure, either from gauze or manual compression of the uterus, but highlight rapid cessation of bleeding and the ability to discontinue pressure application while maintaining hemorrhage control.^33–36^ The improved adhesive ability from the PDA-gel/nSi hydrogel over FloSeal, which has demonstrated clinical capability in managing PPH, suggests that the PDA-gel/nSi hydrogel may be used in a similar fashion with additional, but temporary, manual pressure applied and that the PDA-gel/nSi hydrogel could be potentially at least as effective in this manner.

Besides adhesion, we desired that the optimized PDA-gel/nSi hydrogel is easily injected to facilitate minimally invasive delivery and seal complex geometries present in the postpartum uterus. Nanosilicates have been used in numerous applications due to their property as a rheological modifier and have frequently been used to impart shear-thinning behavior to hydrogels.^13,37^ To visually demonstrate this property, we constructed a simple benchtop model according to our previous methods^38^ with a narrow channel leading to a larger cavity (Figure 1F). The hydrogel was able to flow easily through an 18-gauge catheter inserted in this narrow channel and was deposited against the back wall of the model’s cavity. The shear-thinning behavior of the PDA-gel/nSi hydrogel was further evaluated in a shear rate sweep at, above, and below physiological temperature (Figure 1G). The hydrogel demonstrated decreasing viscosity with increasing shear rates at all temperatures and a corresponding decrease in the consistency index (Figure S6A). The shear-thinning index, determined from the exponent in a power-law fit of the viscosity-vs-shear rate curve, did not display any significant change over the specified temperature range (Figure S6B), indicating that the hydrogel retained shear-thinning behavior across these temperatures.

We further evaluated the injectability of the PDA-gel/nSi hydrogel by measuring the force required to extrude the hydrogel from a syringe. We quantified the force required for extrusion through 14G (approximately equivalent to 6F), 16G (approximately equivalent to 5F), and 18G (approximately equivalent to 4F) needles to determine the impact of diameter on the force required to extrude the hydrogel (Figure 1H) as well as at flow rates of 1 mL/min, 5 mL/min, and 10 mL/min to determine the impact of flow rate on the force required to extrude the hydrogel (Figure 1I). Unsurprisingly, increasing the flow rate or decreasing the needle diameter required increasing force to extrude the hydrogel. At a flow rate of 1 mL/min, the maximum force required to extrude the PDA-gel/nSi hydrogel was 42.00 ± 9.83 N, 76.81 ± 5.49 N, and 108.24 ± 28.83 N for 14G, 16G, and 18G needles, respectively (Figure S7A). This force stabilized to 33.34 ± 9.91 N for a 14G needle, 67.93 ± 5.14 N for a 16G needle, and 87.88 ± 12.68 N for an 18G needle (Figure S7B). The 14G needle resulted in a plateaued force significantly lower than that of the 16G or 18G needles, and the 18G needle displayed a significantly higher maximum force than the 14G needle. A similar trend appeared for increasing flow rates with a constant 14G needle. At 5 mL/min, the extrusion force peaked at 61.00 ± 14.72 N and stabilized at 54.54 ± 15.74 N, while at 10 mL/min, the extrusion force peaked at 94.37 ± 13.30 N and stabilized at 75.24 ± 12.04 N (Figure S7C-D). Only the 10 mL/min flow rate demonstrated any significant increase over the other flow rates in terms of average plateau force or maximum force. The literature is somewhat ambiguous regarding the desirable force requirements for hand-held injected materials, with upper limits beginning as low as 20 N for clinical applications^14^ or 40 N for hand injection,^13,39^ while higher limits can be found at 71 N for a two-finger pinch motion,^40^ or 135–184 N for downward thumb motions.^14,41^ The maximum forces identified for the PDA-gel/nSi hydrogel remain below the 135 N threshold for all tested needle gauges and flow rates, suggesting that the hydrogel could be delivered at least as rapidly as 10 mL/min. We identified 71 N as a middle-ground threshold to evaluate the injectability of the hydrogel (Figure 1H-I): flow rates up to 5 mL/min are possible when using a 14G needle, but smaller needle sizes are unable to extrude the hydrogel with this low level of force. Larger bore catheters or macrosize tubing may be beneficial for rapid delivery of this hydrogel at even higher flow rates.

### The PDA-Gelatin/Nanosilicate Hydrogel Demonstrated Hemostatic Ability and Appropriate Hemo- and Cytocompatibility

We evaluated the PDA-gelatin/nSi hydrogel for hemostatic ability using whole human blood from only female donors (Figure 2A). The PDA-gel/nSi hydrogel presented a 19.28 ± 10.56% reduction in clotting time, which was significantly improved over Surgifoam (10.01 ± 7.87% reduction) and no treatment but was notably much slower to clot than kaolin (59.67 ± 6.26% reduction). This reduction in clotting time is similar to that seen for another nanosilicate/dopamine-gelatin conjugate which utilized a coinjection in situ gelation strategy (∼2.4 min reduction; ∼19% from negative control)^14^ and to that of a gelatin-methacrylate-catechol hemostatic sealant cross-linked with visible light (∼4.6 min reduction; ∼29% from negative control).^42^

**Figure 2:**
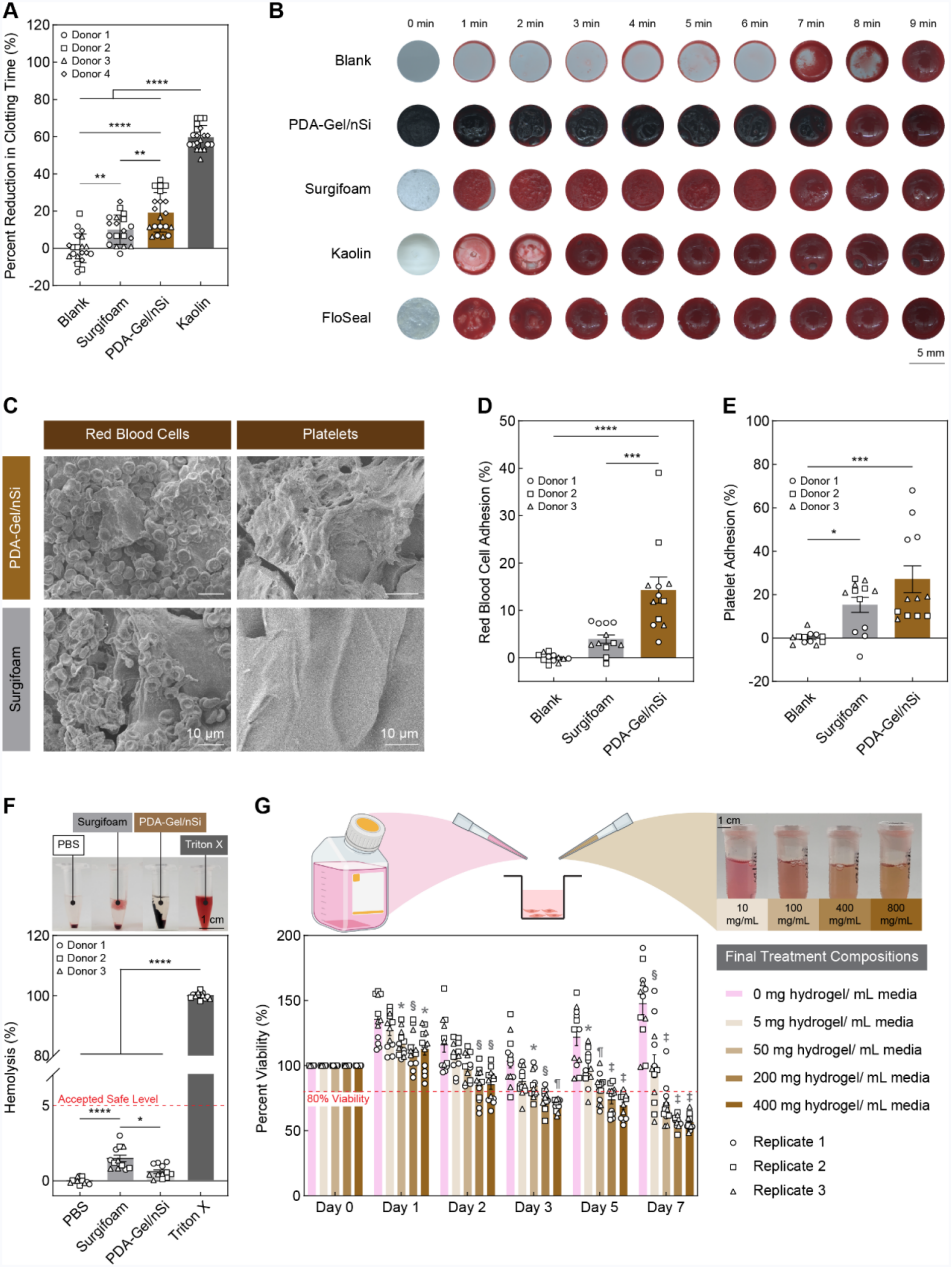
Hydrogel hemostatic ability, interaction with blood cells, and cytocompatibility. A) Whole blood clotting time inversion test with human blood. Clotting time is presented as percent reduction in clotting time to facilitate comparison of results across multiple unique donors. *N* = 19 across 4 unique donors for Surgifoam due to damaged sample (*N* = 4–5 each donor); all other groups have *N* = 20 across 4 unique donors (*N* = 5 each donor). Data shown are mean ± standard deviation. Ordinary one-way ANOVA with Tukey’s multiple comparisons test. *p < 0.05, **p < 0.01, ***p < 0.001, ****p < 0.0001. B) Static well plate clotting assay with bovine blood, visualizing clot formation in contact with blank wells, PDA-gel/nSi, Surgifoam, kaolin, or FloSeal. C) Representative images from scanning electron microscopy (SEM) images of PDA-gel/nSi and Surgifoam following exposure to red blood cells and platelets. Images have been uniformly adjusted for contrast and brightness using Adobe Photoshop for qualitative representation only. Original files and Photoshop files are available in Zenodo repository. D) Quantitative evaluation of red blood cell adhesion. *N* = 12 across 3 unique donors (*N* = 4 each donor). Data shown are mean ± standard error of the mean. Ordinary one-way ANOVA with Tukey’s multiple comparisons test. *p < 0.05, **p < 0.01, ***p < 0.001, ****p < 0.0001. E) Quantitative evaluation of platelet adhesion. *N* = 12 across 3 unique donors (*N* = 4 each donor). Data shown are mean ± standard error of the mean. Ordinary one-way ANOVA with Tukey’s multiple comparisons test. *p < 0.05, **p < 0.01, ***p < 0.001, ****p < 0.0001. F) Quantitative evaluation of hemolytic activity of hydrogel. *N* = 12 across 3 unique donors (*N* = 4 each donor). Data shown are mean ± standard error of the mean. Ordinary one-way ANOVA with Tukey’s multiple comparisons test. *p < 0.05, **p < 0.01, ***p < 0.001, ****p < 0.0001. Representative images of each sample following the 1 h incubation with red blood cells are shown. G) Indirect cytocompatibility determined via Alamar Blue. Percent reduction of Alamar Blue is normalized against percent reduction values from Day 0. Three hydrogel replicates were conducted with 4 wells of cells each; *N* = 12 across three distinct hydrogels (*N* = 4 each hydrogel). Data shown are mean ± standard error of the mean. Two-way ANOVA with Tukey’s multiple comparisons test. Symbols indicate a significant difference compared to control cells for the same time point: *p < 0.05, §*p* < 0.01, ¶*p* < 0.001, ‡*p* < 0.0001.

In addition, we observed hemostatic ability in a static model of clotting using whole bovine blood (Figure 2B). A 96-well plate was coated with samples at the base of the well and incubated with recalcified bovine blood for specified time periods. At each time point, unclotted blood was carefully aspirated and the well was photographed. A similar trend to the inversion test appeared: positive controls FloSeal and kaolin demonstrated rapid clotting within 3 min, followed by slower clotting in the rest of the groups. Surgifoam exhibited an ability to absorb the majority of the blood and retain this absorption at each time point beginning at 2 min, but additional clotting clearly visible above the surface of the Surgifoam sample was not observed until approximately 7 min. PDA-gel/nSi hydrogel displayed clotting at 8 min, which is considerably slower than the positive controls but still represents an improvement over the blank wells which clotted fully at 9 min. We observed a similar trend with this experiment repeated in a 48-well plate (Figure S8), where FloSeal and kaolin achieved the fastest clotting times, followed by Surgifoam and PDA-gel/nSi, with the blank/no-treatment group presenting the slowest clotting. Similar to the smaller well-plate assay (Figure 2B), Surgifoam and PDA-gel/nSi displayed clotting times in sequence, although a much larger stratification of all noted clotting times was present with the larger blood volume and well plate. Overall, both the dynamic inversion test and static well-plate clotting visualization indicate similar trends wherein the PDA-gel/nSi hydrogel exhibits faster clotting than blank setups.

In addition to exploring overall clotting time, we hypothesized that the presence of polydopamine within the PDA-gel/nSi hydrogel would improve the ability to aggregate and adhere to blood cells, thus facilitating hemostasis through mucoadhesive hemostatic action. After incubation with a solution of red blood cells and separately with a solution of platelets, we observed adhesion of red blood cells and platelets to the PDA-gel/nSi hydrogel under scanning electron microscopy (SEM) (Figure 2C). We did not observe any obvious differences between PDA-gel/nSi and Surgifoam regarding adhesion of red blood cells when examined using SEM as an abundance of cells were present on each sample. However, when red blood cell adhesion was quantitatively evaluated (Figure 2D), we noted significant improvement for PDA-gel/nSi over the other groups. In this case, the adhered cells were lysed with Triton-X detergent to release hemoglobin for colorimetric detection at 540 nm. Background absorbance from polydopamine (Figure S9A) was subtracted out to determine the absorbance due to only red blood cells. PDA-gel/nSi demonstrated 14.29 ± 9.52% adhesion of red blood cells, representing a significant improvement over the red blood cell adhesion of all other groups (Surgifoam: 3.96 ± 3.00%; blank: 0.00 ± 0.90%) (Figure 2D). Platelet adhesion was similarly quantified by incubating samples with a solution of isolated platelets, washing the samples, and lysing the adhered cells. A lactate dehydrogenase (LDH) assay kit was utilized to quantify the release of LDH from the adhered cells for detection at 490 nm. Background absorbance from polydopamine (Figure S9B) was subtracted out to determine the absorbance due to only LDH released from adhered platelets. A similar trend was observed regarding adhesion of platelets: PDA-gel/nSi displayed the highest percentage of adhered platelets (27.07 ± 21.23%), which was significantly higher than that of blank tubes (0.00 ± 2.61%) (Figure 2E). However, PDA-gel/nSi did not present a significant difference from Surgifoam (15.30 ± 12.00%), although a numerical increase was present. From these results, it is clear that PDA-gel/nSi hydrogel is able to improve adhesion of both red blood cells and platelets, suggesting a potential mechanism for the hemostatic action of this material.

We further explored how the PDA-gel/nSi hydrogel would interact with blood and other cells to gain an understanding of the material’s hemocompatibility and cytocompatibility. Hemocompatibility was evaluated using an in vitro comparative hemolysis test based on guidance from ISO 10993–4 and ASTM F576–17. Samples were directly exposed to a solution of red blood cells, and the percentage of hemolysis was quantified relative to positive and negative controls (Figure 2F). Hemolysis was quantified by incubating the samples with red blood cells and subsequently measuring the absorbance at 540 nm, corresponding to hemoglobin, to determine the percentage of red blood cells lysed by contact with the sample. Background absorbance from polydopamine was again subtracted out. We observed strong hemocompatibility among all samples tested: PDA-gel/nSi displayed hemolysis of 0.60 ± 0.12%, which was not significantly different from PBS (0.00 ± 0.06%), and represented a significant decrease from Surgifoam at 1.48 ± 0.21% (Figure 2F). This level of hemolysis falls below the generally accepted limit of 5%, indicating that the hydrogel interacts appropriately with blood cells. Further, Surgifoam is a clinical product used in an off-the-shelf format, so the similar hemolysis level observed in PDA-gel/nSi suggests good hemocompatibility with clinical potential.

Prior to performing cytocompatibility testing, we ensured adequate sterilization of the polymer. We first sterilized the PDA-gelatin polymer under ultraviolet (UV) light and subsequently dissolved the sterile polymer into complete endothelial cell media (ECM media) to prepare “leached media” (Figure S10A). This leached media was then aliquoted into the wells of a tissue-culture plate without any cells. We performed an Alamar Blue assay on days 0 and 7 to evaluate for any bacterial growth. As there was no significant difference observed in the percentage reduction for either regular media or in media containing the sterilized PDA-gel (Figure S10B), we concluded that there was no bacterial growth present from the sterilized polymer. We also checked the FTIR spectra of the polymer before and after sterilization (Figure S10C) and did not observe any changes to the peaks present in the spectra. Finally, we evaluated the mechanical strength of the hydrogel with and without UV sterilization (Figure S10D) and observed no significant differences in the onset point of the hydrogel before or after sterilization.

General cytocompatibility was assessed with indirect exposure of the PDA-gel/nSi hydrogel to human umbilical artery smooth muscle cells (SMCs). This cell line was selected in consideration of smooth muscle exposure following detachment of the placenta and the targeted application of the hydrogel. Sterile hydrogel samples were prepared and submerged in complete media at a ratio of 400 mg hydrogel per mL of media. After 24 h at 37 °C, the media was centrifuged to remove solid particulates, and the supernatant was used to expose cells to material leached from the hydrogel. We exposed cells at concentrations of 100%, 75%, 50%, 25%, 10%, 1%, and 0% leached media with the balance being regular media. These mixtures correspond to final hydrogel ratios of 400 mg/mL, 300 mg/mL, 200 mg/mL, 100 mg/mL, 40 mg/mL, 4 mg/mL, and 0 mg/mL, respectively. We observed no significant change in viability after 1 day for cells exposed to up to 50% leached media in comparison to control cells (Figure S11) but did observe a decrease in viability beginning with exposures of 75% leached media and above. Previous studies have noted that nanosilicates are able to sequester proteins^43,44^ and our earlier study with a nanocomposite shape memory foam noted a similar drop in viability for cells exposed to 100% leached media produced from media exposed to the nanocomposite foam.^45^ We hypothesized that nanosilicates present in the hydrogel were sequestering the essential proteins and other nutrients from the cell culture media, resulting in decreased viability for high concentrations (i.e., 75–100%) of leached media.

To gain a more complete understanding of the PDA-gel/nSi hydrogel’s cytocompatibility, we conducted a similar indirect exposure assay with a constant dilution of leached media and varying initial hydrogel to media ratios (Figure 2G). We prepared leached media with an initial hydrogel to media ratio of 10, 100, 400, and 800 mg of hydrogel per mL of media. The leached media was prepared using the previously described procedure. We exposed cells with a 50:50 volume ratio mixture of leached media and regular media, resulting in final concentrations of 5, 50, 200, and 400 mg hydrogel per mL media, respectively, in order to evaluate the impact of varying hydrogel exposure levels, without the influence of a lack of essential nutrients from the media. Cellular viability was determined using an Alamar Blue assay according to the manufacturer’s instructions and normalized to Day 0 (i.e., pretreatment) cell viability. Over a period of 7 days, we observed that an exposure level of 5 mg hydrogel per mL media maintained cellular viability above 80%. There was no significant difference noted for this concentration over 3 days in comparison to control cells. An exposure level of 50 mg hydrogel per mL media similarly allowed for cell viability above 80%^15^ through Day 2 but did present a statistically significant decrease compared to the control cells at each time point. Notably, all exposure levels presented good viability demonstrating cellular proliferation on Day 1, suggesting that short-term application of this material may be feasible. Additionally, the addition of hydrogel to the media did not present any significant impacts to the pH of the media (Figure S12), suggesting that short-term exposure may be reasonable. Similar results were observed in a Live/Dead assay (Figure S13), wherein cells were observed to proliferate following indirect exposure to a weakly concentrated leached media (5 mg hydrogel/mL of media) with qualitatively similar results to the control cells (TCPS group). Cells exposed to a more concentrated leached media (400 mg hydrogel/mL of media) exhibited decreased proliferation and a reduced cell population beginning on Day 3. Sparse dead cells were observed, suggesting that dead cells were detached and aspirated during the imaging assay. In all cases, remaining cells exhibited similar, elongated morphology consistent with healthy SMCs. Overall, this demonstrates a consistent result with those observed in the Alamar Blue assay wherein low exposure to the hydrogel results in the best cytocompatibility through 7 days. Further evaluation of the cellular interactions as well as the consequences of protein sequestration should be explored in detail prior to clinical use.

### In Vivo Efficacy of the PDA-Gelatin/Nanosilicate Hydrogel Is Demonstrated in a Rat Liver Biopsy Punch Model of Uncontrolled Hemorrhage

We further evaluated the hemostatic ability of the PDA-gel/nSi hydrogel in vivo using a well-established rat model of uncontrolled hemorrhage. We have previously utilized this model to evaluate expandable hemostatic composite scaffolds^46^ and observed considerable bleeding with a lack of treatment, which would allow for evaluation of the PDA-gel/nSi hydrogel under conditions of uncontrolled bleeding and active blood flow. Briefly, animals were anesthetized to undergo a liver biopsy punch (Figure 3A). A biopsy punch was used to create a 6 mm defect, and the tissue sample cut away with scissors to create an incomplete cavity. The cavity was observed to bleed freely, and exactly one treatment was administered immediately. Blood loss and clotting time were quantified and compared among no treatment, FloSeal, and PDA-gel/nSi hydrogel. The animals were monitored for 1 h and euthanized after this time. FloSeal was selected as a positive control as a comparative injectable material capable of filling cavities in a similar manner to the test material.

**Figure 3:**
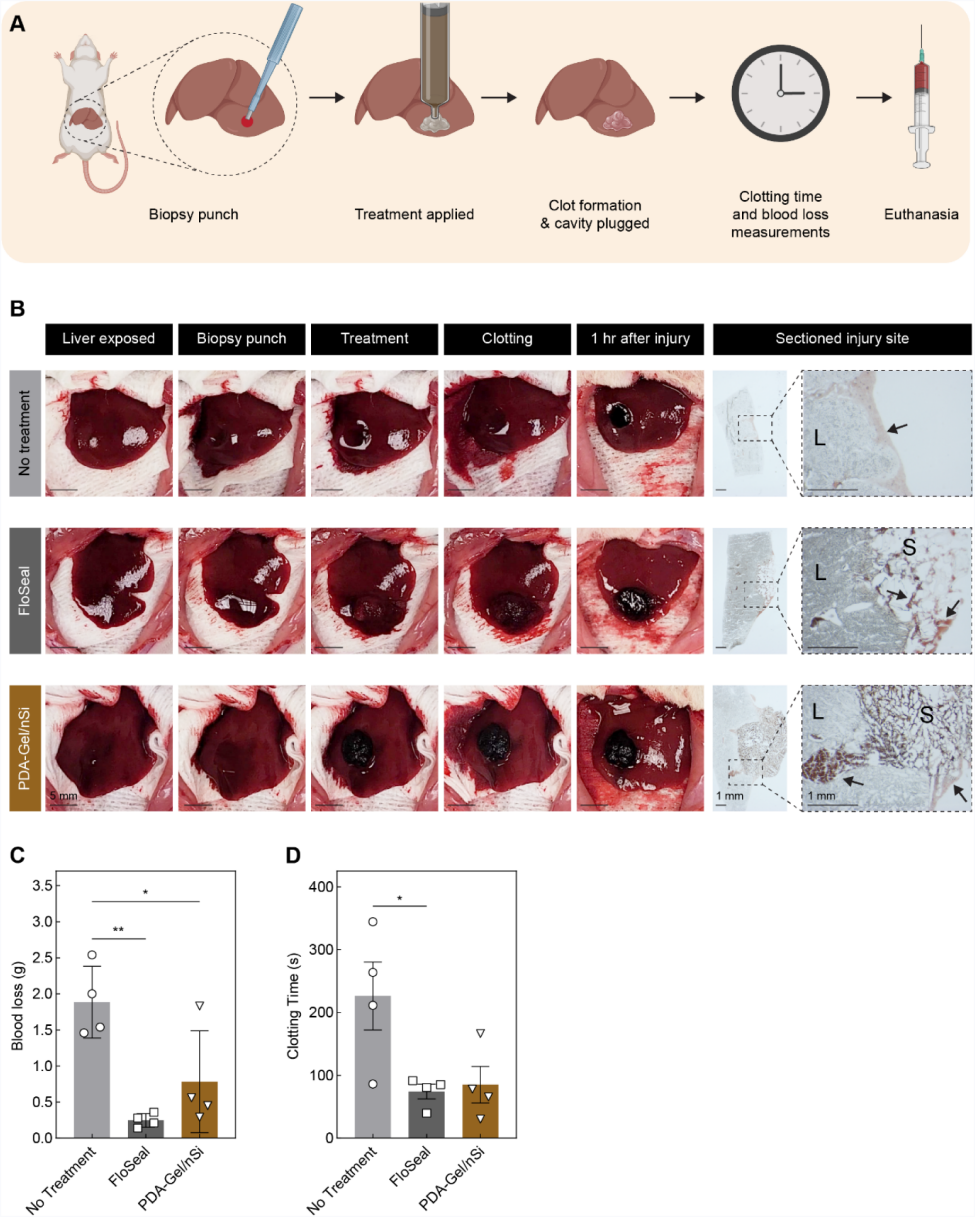
In vivo efficacy in a rat model of uncontrolled hemorrhage. A) Schematic detailing sequence of events for in vivo study. Events noted are biopsy punch creation, treatment applied, clot formation and cavity plugged, clotting time and blood loss measurements, and euthanasia. Schematic was made in part using Biorender.com and Adobe Illustrator. B) Images showing rat liver with the liver exposed, the biopsy punch, treatment applied, clotting, and the liver 1 h after injury, as well as sections from the liver injury site at 1.6X (left) and 8X (right) magnification. *L* indicates the liver tissue, *S* indicates the sample, and the arrows indicate the location of blood clots. Representative images for each group (no treatment, FloSeal, and PDA-gel/nSi) are shown. Brightness and contrast have been uniformly adjusted in Adobe Photoshop to facilitate visualization of surgery images; brightness, contrast, and red color were uniformly adjusted using Adobe Photoshop to facilitate visualization of tissue sections. Original files and Photoshop files are available in Zenodo repository. C) Blood loss measurements determined by change in mass of preweighed gauze. *N* = 4; data shown are mean ± standard deviation. Ordinary one-way ANOVA with Tukey’s multiple comparisons test. *p < 0.05, **p < 0.01, ***p < 0.001, ****p < 0.0001. D) Clotting time measurements determined visually from surgical videos by three independent reviewers. Each point is an average of the determinations made by the three reviewers. *N* = 4; data shown are mean ± standard error of the mean. Ordinary one-way ANOVA with Tukey’s multiple comparisons test. *p < 0.05, **p < 0.01, ***p < 0.001, ****p < 0.0001.

Visualization of the injury site (Figure 3B) revealed the ability to fill the wound cavity with the injectable materials. In each case, the treatment was observed to accumulate on the surface of the liver after filling the biopsy punch cavity. All samples were observed to remain in place after 1 h, suggesting that the mechanical strength of the treatments was sufficient to withstand bleeding at the injury site. Additionally, the samples were noted to become dark red in color, indicating absorption and infiltration with blood. The presence of blood postmortem was observed in tissue sections obtained from the liver injury site (Figure 3B). From these images, clot formation (indicated by the presence of red blood cells in the fixed sample) can be observed both between the sample and the liver tissue as well as mixed in the sample itself and on the surface of the sample. Qualitatively, these results suggest that the injectable materials (FloSeal and PDA-gel/nSi) are each able to plug the tissue cavity and achieve clotting. We observed that all treatment groups resulted in both lower blood loss (Figure 3C) and faster clotting time (Figure 3D) compared to no treatment. No treatment resulted in blood loss of 1.89 ± 0.50 g and is comparable to our previous study.^46^ This was significantly reduced in the presence of both FloSeal (0.25 ± 0.09 g) and PDA-gel/nSi (0.78 ± 0.71 g). There was no significant difference noted between FloSeal and PDA-gel/nSi. A similar trend appeared when examining the clotting time: no treatment required the longest time to stop bleeding, while FloSeal and PDA-gel/nSi both displayed faster clotting times. FloSeal presented a significant decrease from no treatment, while PDA-gel/nSi only presented a numerical decrease (p = 0.0523). However, no significant difference was noted between the clotting times for FloSeal or PDA-gel/nSi, suggesting equivalent efficacy. Although the PDA-gel/nSi hydrogel did not present any significant advantages over the clinical control materials, it did still demonstrate equivalent efficacy in terms of blood loss and clotting time. Therefore, this material may present a viable alternative to clinical materials such as FloSeal, which includes recombinant thrombin and therefore is much more expensive.

### The PDA-Gelatin/Nanosilicate Hydrogel Demonstrates Potential for Assisting in Managing PPH

To better evaluate the PDA-gelatin/nSi hydrogel under relevant physiological conditions, we designed a benchtop uterine model with simulated blood flow from multiple sites (Figure 4A). During pregnancy, the end-supply blood vessels, known as spiral arteries, undergo considerable remodeling and lose their smooth muscle layer to facilitate blood flow to the fetus by way of the placenta.^32^ For the purpose of developing a model, we assumed that bleeding following delivery, i.e., postpartum hemorrhage, would result from the ends of the spiral arteries adjacent to the placenta in the intervillous space which become exposed following detachment and delivery of the placenta. These vessels increase in diameter as they approach the placenta from an initial diameter of approximately 0.4–0.5 mm to a final diameter of approximately 2.5 mm.^32^ Burton et al. previously performed calculations utilizing the Hagen–Poiseuille equation (eq 1, below) to understand the flow occurring between the uterus and the placenta.^32^ In their study, the authors utilized a pressure drop of 80 mmHg to lead their calculations and determined the blood flow velocity in each vessel to be approximately 100–200 cm/s.^32^ However, other studies differentiate between the pressure drop along the proximal portions of the spiral arteries (∼80 mmHg) and the pressure drop into the intervillous space between the uterus and placenta (∼10 mmHg).^31^ Using the same calculation and adjusting the pressure drop to 10 mmHg reveals a blood flow velocity of approximately 22 cm/s. This adjusted velocity is aligned with the peak systolic velocity noted for spiral arteries at the end of pregnancy (∼25 cm/s) determined through a meta-analysis.^47^ As such, we elected to model this flow velocity of ∼25 cm/s in our model to evaluate the hydrogel’s ability to be applied and remain adhered under flow conditions mimicking these vessels following delivery of the placenta.

**Figure 4:**
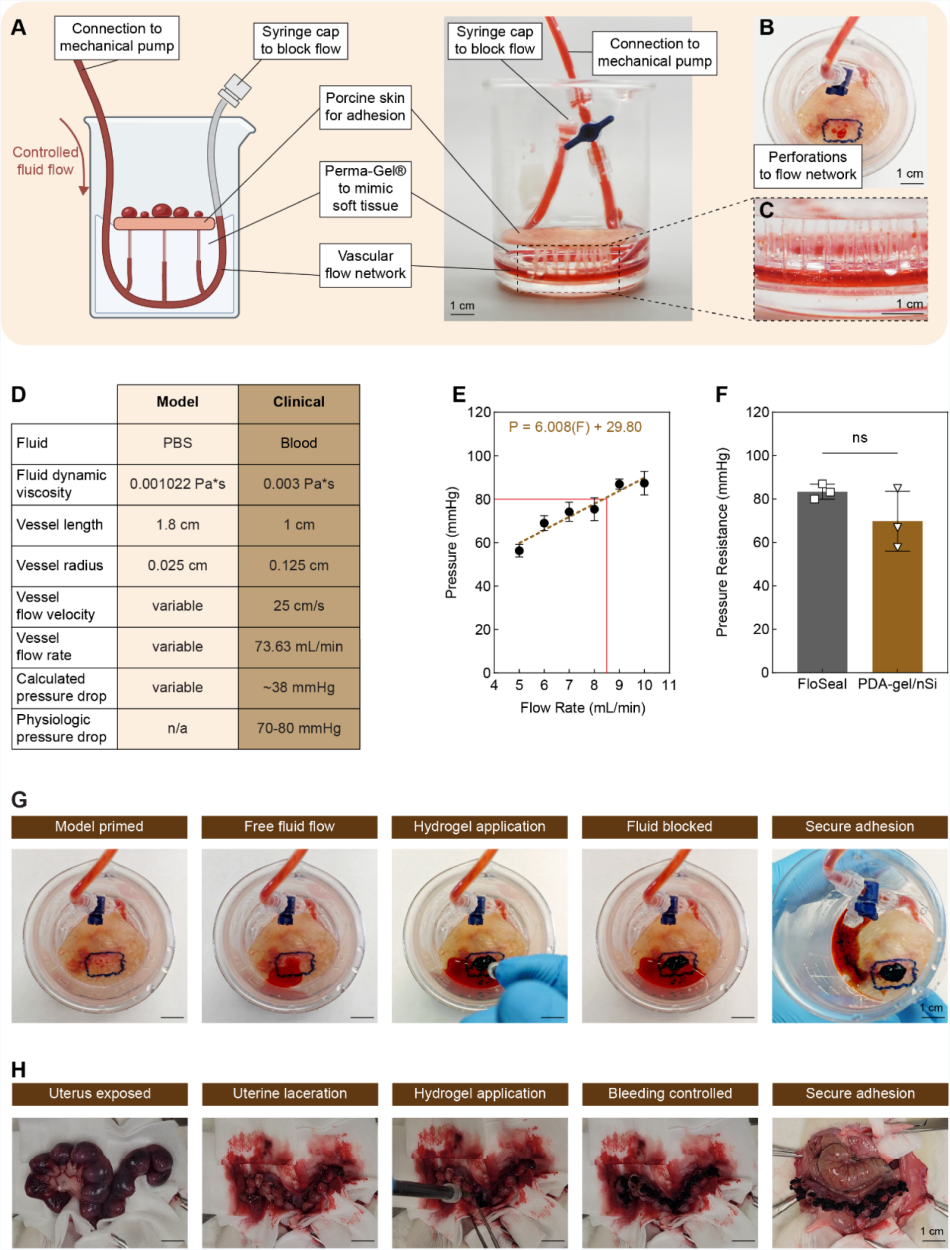
Postpartum hemorrhage treatment proof-of-concept. A) Design of the benchtop model to mimic the postpartum uterus. Key components are indicated: connection to mechanical pump, syringe cap to block flow from large tubing, porcine skin surface for adhesion, Perma-Gel to mimic soft tissue, and vascular flow network. Schematic was made in part using Biorender.com and Adobe Illustrator. B) Aerial view of model highlighting the perforations through porcine skin to connect to the flow network. Dark line indicates the region of the model containing the 27 vertical vessels of the flow network. Red-dyed PBS is shown extruding through the perforations. C) Enlarged photograph of flow network composed of large primary tube and 27 smaller tubes with outlets on the model surface. Flow network is shown without porcine skin on model surface. Red-dyed PBS is present throughout the network to facilitate visualization. D) Comparison of parameters for model fluid calculations. Parameters: fluid used, fluid dynamic viscosity, vessel length, vessel radius, vessel flow velocity, vessel flow rate, calculated pressure drop, and physiologic pressure drop. E) Pressure vs flow rate relationship measured using a digital manometer connected to the model flow network. *N* = 3; data shown are mean ± standard deviation. Linear regression indicates a positive relationship with pressure (*P*) = 6.008 * flow rate (*F*) + 29.80, where pressure is measured in mmHg and flow rate is measured in mL/min. Red lines indicate required flow rate to achieve 80 mmHg pressure. F) Pressure resistance measurements for FloSeal and PDA-Gel/nSi. Pressure is determined as the maximum pressure achieved with sample covering the model outlet holes. *N* = 3; data shown are mean ± standard deviation. Unpaired two-tailed *t* test. *p < 0.05, **p < 0.01, ***p < 0.001, ****p < 0.0001. G) Image sequence showing model primed with red-dyed PBS, free fluid flow through the flow network onto the surface of the model, hydrogel application onto standing fluid, successful fluid blockage, and secure adhesion. The model is tilted in the image showing secure adhesion to highlight hydrogel’s adhesion to the application surface. H) Representative images from rat uterine laceration model. Images highlight uterine exposure, uterine laceration, hydrogel application, bleeding control, and secure adhesion of the hydrogel following gauze removal.

This model was fabricated using commercially available PermaGel, which is a commercial synthetic polymer blend with mechanical properties matched to human tissue. Additionally, PermaGel is transparent, allowing for visualization of flow throughout the flow network and it provides a supportive framework for the model components. The surface of the model was covered in porcine skin to provide a surface to evaluate hydrogel adhesion under flow conditions (Figure 4B). This porcine skin was perforated to connect with a flow network with 27 exit sites (Figure 4C) which provided flow from multiple sites against the applied hydrogel. The flow network was connected to a mechanical system in order to provide controlled and consistent fluid flow through the model. We used microfluidic tubing with an internal diameter of 0.5 mm to model the spiral arteries and adjusted the flow rate to achieve comparable pressure. We utilized red-dyed PBS in our model in order to facilitate testing of the model in nonbiohazard laboratory spaces. To identify the flow rate needed to produce an equivalent pressure drop in the model as in the clinical scenarios, we used the Hagen–Poiseuille equation (eq 1),

1 $$\Delta P=\frac{8\eta LQ}{\pi r^{4}}$$

where η is the fluid dynamic viscosity (blood, 0.003 Pa·s^32^ vs PBS, 0.001022 Pa·s^48^), *L* is the length of the vessel (1 cm, as typically modeled for spiral arteries^32^ vs 1.8 cm, accounting for the length from the start of the vessel to the model surface), *Q* is the flow rate (calculated from the cross-sectional area of the vessel and a velocity of 25 cm/s,^47^ vs determined via this calculation), and *r* is the radius of the vessel (0.125 cm, from a diameter of 2.5 mm^32^ vs 0.025 cm for the model microfluidic tubing), and we assume that the vessels are perfect cylinders of constant diameter; we determined that the desired flow rate in our model would be approximately 5.19 mL/min (Figure 4D).

To gain a better understanding of the actual pressure in the model, we connected a digital manometer to the flow network and monitored the pressure for varying flow rates between 5 mL/min and 10 mL/min (Figure 4E). In contrast with our calculations, we observed that a flow rate of ∼9 mL/min was required to achieve a pressure above 80 mmHg (Figure 4E), which produces a relevant condition for PPH. We then used this flow rate to evaluate the maximum pressure resistance from the PDA-gel/nSi hydrogel and from FloSeal and quantitatively compare the pressure resistance capability between these materials. Each material was applied onto a wetted surface and red-dyed PBS was pumped through the model until the sample was observed to leak or detach. FloSeal presented a slightly elevated pressure resistance PDA-gel/nSi at 83.5 ± 3.5 mmHg, but was not statistically significant from PDA-gel/nSi at 69.9 ± 13.8 mmHg (Figure 4F). These results indicate that the PDA-gel/nSi hydrogel is able to provide comparable pressure resistance to a clinical material (i.e., FloSeal) under relevant pressures and is comparable to the 70–80 mmHg pressure observed in the spiral arteries. Notably, the pressure levels observed in the model testing are considerably higher than the results obtained from the burst strength testing (Figure 1D-E). It is important to note that the burst strength test setup included a defect with a 3 mm diameter, which is much larger than the individual outlet holes present in the model with diameters of approximately 0.5 mm. The relatively higher presence of tissue surrounding the defects may have contributed to the higher bursting pressures observed with the model and suggests that the hydrogel will be better suited for covering small, end-supply blood vessels rather than large vessels.

In a separate experiment, we used a flow rate of 5 mL/min to evaluate the hydrogel’s adhesion under lower flow conditions (Figure 4G) to aid in visualization of the hydrogel’s adhesion as well as consider the use of the hydrogel as a secondary adjunct in managing refractory PPH with a leaking-type bleeding.^33–35^ We observed that the hydrogel could be applied even as more fluid is accumulating and that the hydrogel was able to stop additional flow despite the continued mechanical pumping of fluid at this lower pressure. We further evaluated the force generated in the model at this flow rate (Figure S14A-B) and identified that a flow rate of 5 mL/min would achieve a stabilized force of 34.26 ± 2.09 N. Interestingly, for 27 vessels each with a cross-sectional area of approximately 0.0020 cm^2^, this equates to a pressure of 48465.1 ± 2962.5 mmHg, well above the physiologic pressure that can be expected and a very inflated value compared to the pressure measured using the digital manometer. Further discussion on this point is present in the Limitations section. Overall, these results indicate that the PDA-gel/nSi hydrogel has the potential for application to bleeding vessels when used in a similar manner to FloSeal for the management of PPH.

In addition to our custom benchtop model, we also sought to evaluate the hydrogel in an in vivo rat model of PPH. Although rats do not experience PPH naturally,^3^ cesarean-section-style uterine lacerations have previously been used to artificially create this condition.^49,50^ We modified our previously conducted liver laceration study^46^ to perform uterine laceration on midterm pregnant rats followed by removal of the fetuses and placentas (Figure 4H). The uterine laceration model provides an opportunity to evaluate the hydrogel’s application to relevant anatomy, which provides an improvement over the anatomy present in the liver biopsy punch model. Due to the multiple placentation sites present in the rat uterus and the structure of the uterus around the implantation site, the rat uterus presents a similar cavity structure compared to that created in the liver biopsy punch model. The PDA-gel/nSi hydrogel was administered directly onto the surface of the uterus where the placentas had previously been attached. Although we visually observed bleeding to cease with application of the hydrogel, there was no significant difference in blood loss between untreated rats and rats treated with the hydrogel (Figure S15A-B). Furthermore, the blood loss per kilogram of body weight measured was considerably lower than expected: the average blood loss for untreated rats was 8.96 ± 0.57 g (Figure S15A), equating to an average of 34.62 ± 1.94 g/kg (Figure S15B), and the average blood loss for hydrogel-treated rats was 7.79 ± 1.32 g (Figure S15A), equating to an average of 29.99 ± 4.65 g/kg (Figure S15B). Progesterone was also administered as a pretreatment in an attempt to increase the blood flow and in turn a more severe case of bleeding; however, animals treated with progesterone only experienced blood loss measured at 9.22 ± 2.06 g (Figure S15A), equating to 33.38 ± 4.64 g/kg (Figure S15B), which did not present a significant change from the no-treatment group. Despite the lack of appreciable differences, we were able to observe application of the hydrogel directly to in vivo tissue and visual identification of hemostasis. Further, the hydrogel remained in place for up to 1 h, including during and following removal of the gauze placed around the uterus, suggesting that this material would be able to maintain hemostasis in a physiological setting.

Besides desiring rapid application of the PDA-gel/nSi hydrogel and good adhesion and hemostasis in the bleeding uterus, it is also desirable to have a material that is easy to remove following successful clinical management. In alignment with the small increase in absorbance observed previously (Figure S9), we observed only minor swelling of the hydrogel when exposed to physiological solutions indicating physiological stability in these environments.^14^ In the presence of PBS, the PDA-gel/nSi hydrogel exhibited a swelling ratio of 1.26 ± 0.09, which was statistically similar to the swelling ratio in human plasma (1.18 ± 0.07) and in whole human blood (1.20 ± 0.10) (Figure S16). However, when exposed to deionized water, the hydrogel exhibited significant swelling at a ratio of 3.47 ± 0.53 (Figure S16). This suggests that deionized water may be useful as a solution to facilitate gentle washing and removal of the hydrogel from the uterus.

We observed similar results when examining the biodegradation of the PDA-gel/nSi hydrogel. We submerged the PDA-gel/nSi hydrogel in solutions of 20% hydrogen peroxide (H_2_O_2_), 0.1 M sodium hydroxide (NaOH), and 5 U/mL collagenase to evaluate degradation in an accelerated degradation study quantified through measuring changes in wet mass. Because each of these solutions was prepared in deionized water, we observed considerable swelling of the material during the initial time points of the study. One factor contributing to this swelling is the presence of nanosilicate, which is known to adsorb water. Under accelerated oxidative conditions, the hydrogel exhibited swelling through 48 h, followed by a steady decline in mass until reaching a negligible mass after ∼19 days (Figure S17A). In hydrolytic conditions, swelling was noted for a much longer period of ∼144 h with a sharp decline in mass to reach a negligible level after only ∼10 days (Figure S17B). A similar swelling period was observed in enzymatic conditions, with wet mass increases noted for ∼120 h followed by a similarly rapid decline to negligible mass levels within ∼8 days (Figure S17C). Sodium hydroxide generates a basic environment for hydrolytic reactions that are responsible for bond cleavage, while collagenase is responsible for breaking down gelatin that is present in the hydrogel. In these cases, the fragmentation of longer polymer chains into shorter segments may have contributed to the additional swelling of the hydrogel compared to the oxidative condition. However, hydrogen peroxide generates reactive oxygen species that are capable of degrading organic polymers, so this phenomenon should be explored further. The presence of polydopamine may also be a factor in the shorter swelling period observed in the oxidative conditions compared to the hydrolytic or enzymatic conditions. Overall, a combination of swelling and biodegradation results in rapid mass loss of the hydrogel and may be an avenue for facilitating removal of the material.

### Future Directions

It is evident that designing effective biomaterial treatments for PPH faces many challenges associated with the unique clinical presentation of PPH in comparison to general hemorrhage. Roughly two decades ago, developments in PPH management centered on updated guidelines, patient care algorithms, and education of clinical personnel.^51,52^ An emphasis appeared on quickly and accurately quantifying and managing blood loss, particularly in the context of administering blood transfusions,^51^ and remains a key intervention in current literature.^18^ Surgical interventions and techniques, including use of topical hemostatic agents especially in the case of cesarean sections, have also been noted as essential with improved techniques and use of new hemostatic materials.^18^ While these remain important components of PPH management, increasing research has been conducted on identifying additional therapeutic agents for medical management,^53^ including carbetocin, misoprostol, sulprostone, tranexamic acid, fibrinogen concentrate or cryoprecipitate, and recombinant activated factor VII, among others.^18,53^ Intrauterine tamponade devices have also seen increasing use as they are able to provide compression in a uterus-sparing method and are recommended as temporary hemostatic measures prior to surgical intervention; however, the evidence for these devices is relatively low and warrants further investigation.^18^ Uterine balloon tamponade has been noted as a technique capable of temporarily managing bleeding;^18,53^ however, additional adjunct treatments are required to definitively manage the bleeding and its underlying cause. Additionally, recent research has focused on the management of PPH in particular for cesarean sections, which present further challenges due to the open surgical area and general procedures to control hemorrhage prior to closing the abdomen which limits certain techniques.^18^ Increasingly, combinations of intravenous therapeutics, manual compression, hemostatic materials, and surgical intervention are used to manage PPH in sequence or in tandem.^54^ These combinations of interventions are often referred to as “bundles” and utilize a variety of approaches to manage PPH.^54^ As the management of PPH continues to evolve, it is likely that combination products capable of performing both pharmacologic as well as physical interventions will have a strong role.

To this end, we have considered the potential for adding oxytocin to the PDA-gel/nSi hydrogel described herein in consideration of future development of this material. We have demonstrated the ability to load oxytocin onto the nanosilicate component of the PDA-Gel/nSi hydrogel, evidenced by dynamic light scattering showing increased size (Figure S18A) and more positive zeta potential (Figure S18B) of the oxytocin-nanosilicate complex. Further, we demonstrated that the addition of oxytocin at 67 μg/mL (equivalent to 40 IU per mL of hydrogel, which is relevant to the high end of oxytocin doses given in current clinical management^8^) and a higher concentration of 1 mg/mL does not adversely affect the clotting ability (Figure S18C) or rheological properties of the hydrogel (Figure S18D-F). We noted that the oxytocin-loaded hydrogels at both concentrations continued to exhibit shear-rate-dependent decreases in viscosity (Figure S18D). The loaded hydrogels did not exhibit any significant changes to the consistency index compared to the unloaded hydrogel, although some variability was noted between the two concentrations (Figure S18E). Similarly, although some numerical differences were noted, no significant differences were seen in the onset point (Figure S18F), indicating that the presence of oxytocin does not interfere with the hydrogel’s mechanical properties. These results suggest that the PDA-Gel/nSi hydrogel may be able to be complexed with additional therapeutic molecules aimed at resolving uterine atony or other underlying conditions and highlight the potential to continue modifying the hydrogel’s composition.

Besides adding additional molecules, the chemical composition of the hydrogel may be modified further to produce better results. Literature indicates that the best material-tissue adhesion occurs when the interfacial energy of the tissue matches that of the biomaterial,^17^ so the hydrogel could be further optimized to provide better adhesion with a different concentration that maximizes this energy match. Adjusting the material composition ratios may also enable better exposure of the nanosilicate to blood, which would be expected to improve the hemostatic ability. Additionally, tough hydrogels are abundant in the literature and touted for their superior adhesion capability,^27,55,56^ which is another potential avenue to improve adhesion of the hydrogel to the uterine surface and to continue developing improved treatments for PPH. In addition to modulating the chemical composition and material ratios, a different form factor of the hemostatic hydrogel may prove advantageous. The delivery mechanism into the uterus (i.e., through a catheter) is limited by the need to determine the sites of bleeding, which may be nearly impossible in the case of PPH. A sprayable form^57^ or integration with the surface of a balloon tamponade device^58^ may improve the ability of the hydrogel to be widely and uniformly applied to the uterus without specific identification of the bleeding sites.

### Limitations

The described studies present several limitations. First, the burst strength of the PDA-gelatin/nSi hydrogel was recorded at 26.1 ± 6.5 mmHg with PBS and 30.5 ± 20.1 mmHg with blood. This burst strength is above the 10 mmHg expected pressure drop present in intervillous space between the uterus and the placenta,^31^ but does not reach the level of the pressure drop over the length of the spiral arteries supplying this blood (∼70–80 mmHg).^31,32^ Additionally, leakage was noted to occur through the samples, rather than at the interface of the sample and the porcine skin, indicating that the PDA-gel/nSi hydrogel exhibits cohesive failure rather than adhesive failure.^30^ This cohesive failure can also help explain the much higher burst strengths observed for patch-style hydrogel adhesives (∼180–320 mmHg)^27,59,60^ or hydrogel systems exhibiting in situ gelation (∼200–600 mmHg),^61–63^ rather than pregelled, injectable hydrogel adhesives detailed in this and in similar studies.^15,29^ Based on these results, it is likely that manual pressure or another adjunct treatment would be required to successfully manage the pressurized blood flow observed in PPH.

When conducting clotting studies, the sex of the donor (all female human donors in this study) can impact the results. There is some evidence that hormone shifts during a menstrual cycle can cause a hypercoagulable state, resulting in a decreased clotting time overall.^64^ This phenomenon could explain the relatively low clotting time reduction percentages observed when working with female human blood donors seen in Figure 2A. Blood collection from postpartum women should be considered in future studies to further probe this effect.

We elected to use Surgifoam as a gelatin-based clinical control instead of FloSeal for some hemostatic assays due to its solid scaffold form factor, as we had previously experienced inconsistent results when using viscous liquid samples in the inversion-style clotting test. Additionally, FloSeal samples were observed to disintegrate when incubated with solutions at 37 °C such as for red blood cell adhesion testing, necessitating the use of Surgifoam instead. The well plate assay, conducted with bovine blood (Figure 2B), revealed clotting times which are longer than the typical times desired for rapid hemostasis; however, it is important to note that bovine blood typically takes longer to clot than human blood,^65^ and so the improvement over no treatment is the more notable conclusion. This same experiment in a larger well plate (Figure S8) presented notably higher clotting times, which may be due to the increased mixing needed to fully recalcify the blood and to form a clot throughout the fluid. However, the increased stratification of the numerical values allows for better distinguishment between the clotting times of the various samples.

We suspect that the differences in adhesion of platelets between the samples observed visually through SEM were limited due to the inherent difficulty of locating cells within a macroscale image, as well as the possibility that the adhered cells were located on the outer surface of the sample, which was difficult to image effectively due to sample positioning on the SEM sample stub. Additionally, we monitored the absorbance of PDA-gel/nSi samples which underwent the red blood cell and platelet adhesion test procedure without exposure to red blood cells or platelets to determine if the dark coloring of the PDA-gel/nSi hydrogel impacted these absorbance-based measurements. We did note a minor increase in the absorbance of solutions exposed to PDA-gel/nSi compared to PBS (Figure S9A-B), which was attributed to the presence of polydopamine. At 490 nm, relevant to the platelet adhesion study, this absorbance increase is ∼0.01133 and at 540 nm, relevant to the red blood cell adhesion study, this absorbance is ∼0.005652. The values discussed for these tests include subtraction of this PDA-gelatin-driven absorbance from the overall absorbance of the sample in order to identify the absorbance attributable to the adhered cells in the form of hemoglobin for red blood cells and LDH for platelets.

During the in vivo rat liver biopsy punch study, application of the treatment did displace some blood, which may have influenced the blood loss measurements. The liver biopsy punch model was selected as a model of uncontrolled, severe hemorrhage due to the abundance of literature on this model and its frequent use in evaluating biomaterials for noncompressible hemorrhage.^46,66^ Although the rat liver biopsy punch model does present uncontrolled, noncompressible hemorrhage, the flow rates and blood volumes are still much lower than what would be present in a human patient. A large animal model would be necessary in order to fully evaluate any added benefit from the wet tissue adhesion. In addition, the liver biopsy punch injury typically produces bleeding that is slower and more consistent with venous-type bleeding, rather than arterial-type bleeding, limiting the efficacy of evaluating a hemostatic material under pressurized flow. Although still a small anatomical size, a rat femoral artery laceration^67^ or rat cardiac puncture model^56^ would allow for evaluating pressurized flow while maintaining the accessibility and lower cost of a small animal model. Further, the shallow defect created in the liver is not representative of the uterine anatomy or uterine blood vessels, which limits the translation of these results. This model does achieve better bleeding conditions in comparison to the uterine laceration model (discussed below) but still does not completely recapitulate the clinical presentation. Similar to the need for large blood flow, use of a large animal model such as a sheep, cow, or nonhuman primate would provide better anatomical comparison.^3^

In our benchtop uterine model, we evaluated the force generated for varying flow rates through this model (Figure S14A-B) and identified that a flow rate of 5 mL/min would achieve a stabilized force of 34.26 ± 2.09 N, corresponding to a pressure of 48465.1 ± 2962.5 mmHg. As this pressure is much higher than physiologically relevant, we expect that the model’s construction limited its flow. It is possible that some of the vessels were inadvertently sealed off, which would cause the force measurement, and consequently the pressure measurement, to be inflated compared to the theoretical calculation. Even a flow rate of 1 mL/min, which presented force of 15.54 ± 1.53 N, would still convert to a pressure of ∼300 mmHg, well above physiological pressures. Additionally, the Δ*P* pressure drop calculated from clinical parameters (Figure 4D), including a flow velocity of 25 cm/s and a vessel radius of 0.125 cm, is approximately 38 mmHg, which is higher than the initially sourced 10 mmHg, highlighting the considerable role of the vessel size in the pressure. If the model’s vessels were inadvertently smaller than the theoretical radius, this would contribute to the inflated force measurements observed in the model. Direct testing with a digital manometer revealed similar issues with the pressure calculation, as the pressure was measured at roughly 50–90 mmHg (Figure 4E) for flow rates between 5 and 10 mL/min. While these pressures are much closer to physiologically relevant pressures, they are notably different than the calculated pressure based on the force measurements. As such, there may have been considerable pressure losses unaccounted for throughout the model due to the geometry of the flow network or other components. The model calculations do not consider the influences of the tubing junctions within the flow network (Figure 4B) and so further detailed calculations would improve this evaluation. In addition, the surface of the model was covered in porcine skin, which is an approximation of human tissues but lacks a complete mimicry of the postpartum uterus and poses potential for inadvertent sealing of vessels or risks leaking gaps present in the final structure.

The uterine laceration model also presented challenges, namely the lack of a clearly hemorrhagic condition. In each treatment group, the average blood loss is much lower than the estimated blood volume for a pregnant Sprague–Dawley rat at roughly 120 mL/kg body weight,^68,69^ suggesting that the uterine laceration model is ineffective to reliably create a truly hemorrhagic condition. The blood flow was noted to be a slow, leaking type flow, which is not representative of the human PPH condition. Additionally, large volumes of amniotic fluid were observed to soak into the preweighed gauze, which would have confounded the blood loss measurement results. Further, the rat uterus does not provide a strong visualization of contractions. However, some contractile activity is able to be seen when viewing sped-up videos taken during the surgeries (Supporting Information Videos 1–3), which provides an advantage over the liver biopsy punch model. In the videos, it is possible to observe contractions and movement of the uterine tissue and that the PDA-gel/nSi hydrogel remains adhered to the tissue surface, suggesting the potential for secure attachment to contracting tissues. However, we acknowledge that the contractile activity is very low in comparison to contractions observed in human patients, and so further testing would be required to validate this point. Further, we are unable to use this model to assess the impact of the hydrogel treatment on mortality as only one of the animals in the progesterone group exhibited mortality. None of the animals in either the no-treatment group or the PDA-gel/nSi hydrogel group exhibited mortality, making it impossible to determine if the hydrogel improves mortality outcomes. This is a major limitation of this model and we attribute the lack of mortality to the overall low bleeding and nonhemorrhagic condition observed following uterine laceration. Alternative animal models such as sheep, which provide larger blood volume, faster blood flow, and a closer uterine anatomical equivalent^3^ should be utilized in the future to fully understand the utility of the PDA-gel/nSi hydrogel. These large animal models can also be used to create a fatal hemorrhagic condition, which would allow for a true assessment of the impact of the treatment.

## Conclusion

We have presented a multifunctional hemostatic hydrogel with adhesive, hemostatic, and hemo- and cytocompatible properties designed to address current clinical challenges in postpartum hemorrhage. This hydrogel demonstrates improved adhesion over a clinical control while retaining injectability and has demonstrated a notable reduction in clotting time in vitro compared to no treatment. Although the PDA-gel/nSi hydrogel does not present significant advantages in terms of hemostatic ability compared to clinical controls, this alternative hydrogel does demonstrate equivalent efficacy in terms of blood loss and clotting time in vivo, suggesting potential as an adjunct treatment for managing PPH. Additional characteristics, including significant swelling in the presence of deionized water and strong short-term hemo- and cytocompatibility, suggest that this material may prove advantageous in certain circumstances. The limitations in terms of adhesive ability may be addressed by using this hydrogel in combination with a manual treatment or mechanical device, thus improving the patient experience and accelerating PPH management.

## Materials and Methods

### PDA-Gelatin Synthesis

Three distinct procedures were considered for conjugation of polydopamine to gelatin (PDA-gelatin V0, V1, and V2).

#### PDA-gelatin V0

Two g of gelatin (300 bloom, type A; Sigma-Aldrich, USA) was dissolved in 140 mL of 2-(*N*-morpholino)­ethanesulfonic acid (MES; Thermo Fisher, USA) buffer at 37 °C. Two g of 1-(3-(dimethylamino)­propyl)-3-ethylcarbodiimide hydrochloride (EDC; Ambeed, USA) and 1.7 g of *N*-hydroxysuccinimide (NHS; Sigma-Aldrich, USA) were added sequentially, dissolved, and allowed to react for 20 min. 900 mg of dopamine hydrochloride (Sigma-Aldrich, USA) was dissolved separately in MES buffer and then added to the gelatin solution. The combined solution was allowed to react for 24 h at 37 °C in the dark. After, the reaction solution was transferred into dialysis tubing (Repligen, USA) with a molecular weight cutoff (MWCO) of 6–8 kDa and dialyzed against reverse osmosis water for 4 days. The retained product was then collected, frozen, and lyophilized. Although this synthesis was intended to directly graft dopamine to gelatin, a brown coloring of the synthesized product was noted, indicating that polydopamine had formed throughout the reaction. For this reason, this synthesis is referred to as “PDA-gelatin V0”.

#### PDA-Gelatin V1

Polydopamine was first synthesized in a one-pot reaction adapted from a previously published method^70^ and subsequently conjugated onto gelatin using EDC/NHS chemistry. 190 mg of dopamine hydrochloride was added to a solution composed of 58 mL 95% ethanol (Decon Laboratories, Inc., USA), 131.5 mL deionized water, and 0.5 mL 1 N sodium hydroxide (NaOH; VWR, USA) and allowed to react for 24 h at 37 °C in the dark to produce polydopamine. After 24 h, the gelatin/EDC/NHS solution was prepared as described in the PDA-gelatin V0 synthesis. The two solutions were combined and allowed to react for 24 h at 37 °C in the dark to enable grafting of polydopamine onto gelatin. After, dialysis and lyophilization were performed as described in the PDA-gelatin V0 synthesis.

#### PDA-Gelatin V2

The PDA-gelatin V1 synthesis was carried out with the following modifications. 900 mg of dopamine hydrochloride was dissolved in 232 mL 95% ethanol, 526 mL deionized water, and 2 mL 1 N NaOH to produce polydopamine. The pH of the gelatin solution was adjusted to 8 using 1 N NaOH prior to combining with the polydopamine solution, enabling grafting of polydopamine onto the gelatin polymer. All other steps of the procedure were conducted as described above.

### Arnow’s Method

The presence of polydopamine in the PDA-gelatin product was quantified using Arnow’s Method.^26^ Briefly, 50 mg of polymer was dissolved in 1 mL deionized water. The polymer solution was vortexed and heated to ensure a homogeneous solution. 250 μL of the solution was then aliquoted into microcentrifuge tubes (VWR, USA) to prepare replicates for analysis. 250 μL of 0.5 N hydrochloric acid (HCl; VWR, USA), 250 μL of a nitrite-molybdate reagent (10% w/v sodium nitrite (Beantown Chemical, USA) and 10% w/v ammonium molybdate (Alfa Aesar, USA)), and 250 μL of 1 N NaOH were added to each tube in sequence. After each reagent addition, the tubes were allowed to shake at 100 rpm for 5 min prior to adding the next reagent. The tubes were then centrifuged at 500*g* for 5 min to collect particulate matter. The supernatant was pipetted into a 96-well plate and the absorbance was read at 430 nm on a plate reader (Infinite 200Pro M Plex, TECAN, Switzerland; Cytation 5, BioTek, USA). Either gelatin without dopamine or standard synthesis conditions served as a control. A standard curve was prepared by performing the same sequence of steps on native dopamine to calculate the concentration of dopamine present in the polymer. In Figure S1, catechol content is normalized to the leftmost data set within each graph to facilitate comparisons among the groups.

### FTIR

Lyophilized polymer was analyzed using Fourier Transform Infrared Spectroscopy (Bruker, USA) using a Bruker ALPHA-Platinum device with a diamond Attenuated Total Reflection crystal. Scans were completed from 400 cm^–1^ to 4000 cm^–1^ wavenumbers. Gelatin without dopamine content served as a control for polymer synthesis validation; unsterilized PDA-gelatin served as a control for the sterilization study.

### NMR

Lyophilized polymer was dissolved in heavy water (deuterium oxide; Millipore Sigma, USA) at a concentration between 15 and 20 mg/mL. Approximately 700 μL of the solution was added to a nuclear magnetic resonance (NMR) tube (DWK Life Sciences, USA) and analyzed via ^1^H NMR using an AVANCE NEO 400 (Bruker, USA) instrument. Data were converted to text format using Bruker TopSpin software (Bruker, USA).

### Hydrogel Fabrication

Hydrogels containing PDA-gelatin and nanosilicates were fabricated at varying concentrations. Briefly, PDA-gelatin was dissolved in deionized water preheated to 37 °C. Separately, nanosilicates (Laponite XLG; BYK Additives, USA) were added to deionized water, vigorously vortexed, and allowed to exfoliate to optical clarity. After, the two solutions were combined, heated to 60 °C to facilitate mixing, and stirred vigorously to produce a homogeneous mixture. Hydrogels were stored at 4 °C for further use. PDA-gelatin-only hydrogels were prepared in the same manner as described above for the PDA-gelatin component.

### Human Blood Samples

Fresh human blood samples were obtained from healthy, consenting donors under Texas A&M University IRB protocol 2022–0501. Blood was collected using standard venipuncture procedures into commercial sodium citrate tubes (Henry Schein, USA) and used the same day. Only female donors were used for this study as this material is intended for use in consideration of postpartum hemorrhage.

### Animal Studies

Ethical approval for use of animals was obtained under the Texas A&M Institutional Animal Care and Use Committee. Approval was granted under Animal Use Protocol #2024–0301 for liver biopsy punch study and under Animal Use Protocol #2024–0262 for pregnant rat use and postpartum hemorrhage rat modeling study. Animals were commercially obtained from any Texas A&M University Comparative Medicine Program-approved animal vendor. Animals were housed for at least 3 days prior to use to allow for acclimation.

### Organ Images

Animal tissues were harvested from a rat immediately postmortem to obtain fresh tissue samples for ex vivo adhesion testing. Tissues were sectioned into pieces to allow for visualization. A hydrogel sample was applied to the tissue and used to adhere the tissue to a nitrile-gloved finger. The tissue was lifted against gravity and photographed to visualize adhesion. Multiple tissues were evaluated: uterus, skin, heart, lung, spleen, kidney, liver, and intestine.

### Weighted Adhesion Lap Shear Style Testing

Pig skin was purchased from the Rosenthal Meat Center at Texas A&M University and stored at −80 °C until use. Pig skin was thawed to room temperature and cut into rectangular sections approximately 1 cm wide and 4 cm long. A 2–4 mm biopsy punch was used to create a hole in the pig skin. A paperclip was threaded through this hole and used to suspend the pig skin in the air. All pig skin sections were soaked in phosphate-buffered saline (PBS) prior to use to provide a hydrated tissue environment. Hydrogel was placed onto one section of pig skin and the mass recorded. This section was then pressed lightly to a suspended tissue section and paperclips were successively added until the lower section fell. The mass of the paperclips and the hanging pig skin was recorded and normalized against the mass of the hydrogel.

### Mechanical Lap Shear Adhesion Testing

Pig skin was purchased from the Rosenthal Meat Center at Texas A&M University and stored at −80 °C until use. Pig skin was thawed to room temperature and cut into rectangular sections approximately 1.5 cm by 3.0 cm × 2.5 mm. Samples were applied to the inside surface of the skin and a second section of skin was applied in an overlapping fashion to create a test specimen. The dimensions of the overlap area were measured using calipers and the mass of the sample was recorded. The test specimen was secured in tension clamps of a mechanical tester (MTest Quattro, ADMET, USA) and pulled apart at a constant rate of 5 mm/min according to ASTM F2255–24. The maximum load was determined and used to calculate the shear strength.

### Burst Strength Testing

Burst strength adhesion testing was conducted using both PBS and blood. A circuit was created wherein a syringe pump fed the specified test solution through tubing at a constant rate. The opposite end of the circuit tubing ended in a section of pig skin which was adhered using superglue to the tubing to prevent any dislodgement. This pig skin section contained a 3 mm circular defect made with a 3 mm biopsy punch to match the diameter of the tubing. The sample (PDA-gelatin/nSi hydrogel, FloSeal, or Band-Aid) was applied to cover the defect. A branch in the circuit connected to a digital manometer to read the gauge pressure generated in real time. The test solution (i.e., PBS or human blood) was pumped into the tubing at a rate of 2 mL/min. All solutions were held at room temperature, and the blood samples were citrated to prevent clotting in the circuit. The manometer was videorecorded and the maximum pressure was recorded as the burst pressure. The sample was observed for any leaking of the solution either through or around the sample.

### Mini Uterus Model Injectability Demonstration

A miniature benchtop model with a narrow channel opening to a larger cavity similar to the uterus was fabricated according to our previously published method.^38^ Briefly, a mold was constructed in SolidWorks (Dassault Systèmes, USA) and 3D-printed using polylactic acid filament (Bambu Lab, China) on a Bambu Lab A1 Mini 3D printer (Bambu Lab, China). A 10% gelatin (Sigma-Aldrich, USA) solution was poured into the mold and allowed to set until firm. The gelatin construct was then submerged in 10% gelatin methacrylate solution and the gelatin methacrylate was cross-linked under ultraviolet (UV) light. After cross-linking, the entire construct was heated to approximately 40 °C to melt the gelatin and washed with PBS, revealing a negative space inside the model. An 18-gauge catheter (Smiths Medical, USA) was attached to a 1 mL syringe (Amazon, USA) filled with the PDA-gel/nSi hydrogel for demonstrating injection of the hydrogel into the cavity.

### Shear Rate Sweep

Shear rate sweep tests were conducted on a DHR-2 rheometer (Waters, USA) using an 8 mm diameter parallel plate geometry and a 0.5 mm gap. A Peltier plate attachment was utilized for temperature control. Tests were conducted at 25 °C, 37 °C, and 45 °C with three replicates per temperature. The shear rate sweep was conducted from 300 s^–1^ to 0.01^–1^ following a 10 min equilibration time.

### Injection Force Test

The force required to extrude the PDA-gel/nSi hydrogel from a syringe was evaluated for varying flow rates (1, 5, and 10 mL/min) and various needle diameters (14, 16, and 18 gauge). Blunt-tip needles (Amazon, USA) were attached to 1 mL syringes (Amazon, USA) filled with the PDA-gel/nSi hydrogel. Syringes were placed in a custom stand to support the syringe in a vertical position. An MTest Quattro mechanical tester (ADMET, USA) was used to depress the syringe at a constant rate and to measure the force generated by this action. Three trials were completed for each condition with clean syringes and needles. For tests comparing various needle diameters, the extrusion rate was set at 1 mL per minute. For tests comparing various extrusion rates, a 14G needle was used.

### Clotting Time Inversion Test

Samples were placed into microcentrifuge tubes. Liquid samples were pipetted into the tubes. Viscoelastic hydrogel samples (e.g., PDA-gel/nSi) were measured by weight and subsequently were centrifuged at 2000*g* for 5 min to collect the material at the base of the tube and provide a consistent surface area across replicates. For kaolin samples, a solution was pipetted into microcentrifuge tubes and allowed to dry in an oven to enable accurate mass measurements. Citrated whole blood and 0.1 M calcium chloride were added to the tube in a 9:1 blood-to-calcium chloride volume ratio and a stopwatch was started. The tube was placed in a heat block to maintain the temperature at 37 °C. Every 15 s, the tube was inverted and observed for flowing. The time when no flowing was observed was recorded as the clotting time. In order to compare clotting time across multiple donors, the percent reduction in clotting time was calculated as follows:

2 $$\mathrm{Reduction in Clotting Time}\left(\%\right)=\frac{\mathrm{Average clotting time of blanks}-\mathrm{clotting time of sample}}{\mathrm{Average clotting time of blanks}}\times 100\%$$

### Static Well Plate Clotting Assay

Samples of PDA-gel/nSi, Surgifoam, kaolin, and FloSeal were applied to coat the surface of individual wells in a 96- or 48-well tissue culture-treated plate (Avantor, USA). To evenly and consistently apply kaolin, a 10% w/v solution of kaolin (Sigma-Aldrich, USA) was prepared in deionized water and pipetted into the well. These wells were allowed to dry fully in an oven before adding other samples. For Surgifoam, a 10 mm (for the 48-well plate) or 6 mm (for the 96-well plate) biopsy punch (Medline, USA) was used to punch circular samples from a sheet of Surgifoam. For PDA-gel/nSi and FloSeal, a combination of spatulas and syringe plunger heads was used to apply the sample across the bottom of the well. Blood and calcium chloride were preheated separately to approximately 38–40 °C to account for some cooling as blood was pipetted into the plate. Citrated whole blood and 0.1 M calcium chloride were added to the tube in a 9:1 blood-to-calcium chloride volume ratio, a stopwatch was started, and the well plate was placed in an oven to maintain the temperature. At specified time points, the well plate was removed from the oven, unclotted blood was carefully aspirated, and the well was photographed using a Discovery V8 SteREO microscope (Zeiss, Germany) at a 1.6× magnification. Individual wells were used for each time point.

### Red Blood Cell Solution Preparation

Fresh, whole, human blood was centrifuged at 1700*g* for 5 min. The supernatant was removed and replaced with 1X PBS. This process was repeated until the supernatant was clear. The pellet was diluted in 1X PBS to produce a 4% red blood cell solution.

### Platelet Solution Preparation

Fresh, whole human blood was obtained and prepared according to Abcam’s “Isolation of human platelets from whole blood” protocol found at: https://www.abcam.com/en-us/technical-resources/protocols/isolation-of-human-platelets-from-whole-blood. Briefly, citrated whole human blood was centrifuged at 200*g* for 20 min to separate blood components. The supernatant was collected as platelet-rich plasma, mixed with HEP buffer (140 mM NaCl (Avantor, USA), 2.7 mM KCl (Sigma Aldrich, USA), 3.8 mM HEPES (Avantor, USA), 5 mM EDTA (Avantor, USA), pH 7.4) and centrifuged again at 100*g* for 20 min to remove contaminating red and white blood cells. The supernatant was again collected and centrifuged at 800*g* for 20 min to pellet platelets. The platelet pellet was washed twice with platelet wash buffer (10 mM sodium citrate (Avantor, USA), 150 mM NaCl, 1 mM EDTA, 1% w/v dextrose (Avantor, USA), pH 7.4) and resuspended in Tyrode’s buffer (134 mM NaCl, 12 mM NaHCO_3_ (Sigma Aldrich, USA), 2.9 mM KCl, 0.34 mM Na_2_HPO_4_ (Sigma Aldrich, USA), 1 mM MgCl_2_ (Avantor, USA), 10 mM HEPES, pH 7.4) with 5 mM glucose (Avantor, USA) and 3 mg/mL bovine serum albumin (Avantor, USA).

### Scanning Electron Microscopy

Samples were placed in a microcentrifuge tube and hydrogel samples (i.e., PDA-gel/nSi) were centrifuged at 2000*g* for 5 min to ensure consistent surface area across samples. Samples were incubated with 0.5 mL of the red blood cell solution or 0.5 mL of the platelet solution at 37 °C for 1 h. Samples were washed with PBS and immersed in a 4% paraformaldehyde (PFA; Thermo Fisher, USA) solution to fix cells. Samples were stored in PFA for at least 24 h to ensure complete fixation. After fixing, samples were washed 3 times with PBS to remove PFA, flash-frozen in liquid nitrogen, and lyophilized overnight. Samples were adhered to imaging stubs using carbon tape and sputter-coated with a platinum–palladium mixture (Sputter Coater 208 HR, Cressington, United Kingdom) prior to imaging. Samples were imaged using a JEOL JSM-7500 cold field emission scanning electron microscope (JEOL, Japan) at 5.0 kV.

### Red Blood Cell Adhesion

Samples were placed in a microcentrifuge tube and hydrogel samples (i.e., PDA-gel/nSi) were centrifuged at 2000*g* for 5 min to ensure consistent surface area across samples. Samples were incubated with 0.5 mL of the red blood cell solution at 37 °C for 1 h. After 1 h, the solution was aspirated and the samples were washed gently 3 times with PBS to remove unadhered cells. A solution of 1% Triton-X 100 (Research Products International, USA) was added to each sample to lyse adhered cells. Samples were incubated with Triton-X for 1 h to ensure complete lysis. Samples were then centrifuged at 2000*g* for 5 min to sediment solid particles and the supernatant was read at 540 nm. The percentage of red blood cells adhered was calculated as

3 $$\mathrm{Red Blood Cell Adhesion}\;\left(\%\right)=\frac{A540_{\mathrm{sample}}-A540_{\mathrm{blank}}}{A540_{\max}-A540_{\mathrm{blank}}}\times 100\%$$

where A540 is the absorbance at 540 nm, blank refers to tubes without any sample, and max refers to tubes with 1% Triton-X 100 as the sample.

### Platelet Adhesion

Samples were placed in a microcentrifuge tube and hydrogel samples (i.e., PDA-gel/nSi) were centrifuged at 2000*g* for 5 min to ensure consistent surface area across samples. Samples were incubated with 0.5 mL of the platelet solution at 37 °C for 1 h. After 1 h, the solution was aspirated and the samples were washed gently 3 times with PBS to remove unadhered platelets. A solution of 1% Triton-X 100 (Research Products International, USA) was added to each sample to lyse adhered cells. Samples were incubated with Triton-X for 1 h to ensure complete lysis. Samples were then centrifuged at 2000*g* for 5 min to sediment solid particles. A 100-uL aliquot of the supernatant was transferred to a 96-well plate and a lactate dehydrogenase (LDH) assay kit (Cayman Chemical, USA) was performed according to the manufacturer’s instructions. The percentage of platelets adhered was calculated as

4 $$\mathrm{Platelet Adhesion}\;\left(\%\right)=\frac{A490_{\mathrm{sample}}-A490_{\mathrm{blank}}}{A490_{\max}-A490_{\mathrm{blank}}}\times 100\%$$

where A490 is the absorbance read at 490 nm, blank refers to tubes without any samples and thus represents the spontaneous release of LDH into the supernatant, and max refers to tubes with Triton-X 100 as the sample and thus represents the maximum LDH release possible for a given volume of the platelet solution.

### Hemolysis

Samples were placed in a microcentrifuge tube and hydrogel samples (e.g., PDA-gel/nSi) were centrifuged at 2000*g* for 5 min to ensure consistent surface area across replicates. Samples were submerged with 1 mL of the red blood cell solution and incubated for 1 h at 37 °C. Samples were then centrifuged at 2000*g* for 5 min and the supernatant was read at 540 nm. Three aliquots from each sample were read. Hemolysis was calculated as follows:

5 $$\mathrm{Hemolysis}\;\left(\%\right)=\frac{A540_{\mathrm{sample}}-A540_{\mathrm{negative control}}}{A540_{\mathrm{positive control}}-A540_{\mathrm{negative control}}}\times 100\%$$

where A540 is the absorbance read at 540 nm, the negative control is tubes with PBS as the sample, and positive control is tubes with Triton-X 100 as the sample.

### Sterilization Study

To confirm the effectiveness of the sterilization procedure, we conducted an AlamarBlue assay without cells using media with dissolved, sterilized polymer. The polymer was sterilized by irradiation under ultraviolet (UV) light (365 nm wavelength) for 30 min and handled under sterile conditions. This polymer was then dissolved in complete endothelial cell media and aliquoted into a 96-well plate. Complete ECM media without hydrogel was used as a control. An Alamar Blue assay (Bio-Rad, USA) was conducted on days 0 and 7 to observe for any change in metabolic activity, indicating bacterial growth in the wells. Five replicates were conducted on polymer samples. Data are reported as percent reduction of Alamar Blue.

### Sterile Hydrogel Preparation

PDA-gelatin and nanosilicates were sterilized under UV light for 30 min with a 365 nm wavelength UV lamp inside the biosafety cabinet (BSC). The “hydrogel fabrication” procedure detailed above was followed with the exception of preparing the components in sterile water and completing the fabrication under sterile conditions in the BSC.

### Stress Sweep

Sterile and nonsterile hydrogels were prepared as described above. Stress sweep tests were conducted on a DHR-2 rheometer (Waters, USA) using an 8 mm diameter parallel plate geometry and a 0.5 mm gap following a 10 min equilibration time. A Peltier plate attachment was utilized for temperature control. Tests were conducted at 25 °C, 37 °C, and 45 °C with three replicates per temperature. The stress sweep was analyzed using the Onset Point analysis function within TRIOS software (Waters, USA). The analysis was performed on the storage modulus data from 20 Pa to the furthest continuous data point.

### Hydrogel Leached Media Preparation

Hydrogel materials (i.e., PDA-gelatin and nanosilicate) were irradiated under UV light at 365 nm for 30 min. The hydrogel was then prepared as previously described using sterile water. Preparation occurred in a biosafety cabinet under sterile conditions. The hydrogel was then added to complete endothelial cell media (ECM media) at 10 mg/mL, 100 mg/mL, 400 mg/mL, and 800 mg/mL final concentrations. The hydrogel-media combination was placed in a dry bath at 37 °C for 24 h. Afterward, the hydrogel-media combination was centrifuged to collect particulate matter and the supernatant was collected into a fresh tube under sterile conditions. This supernatant was stored as “leached media” at 4 °C for further use.

### Leached Media pH Testing Analysis

Media was prepared according to the procedure described above. The pH of each leached media sample was tested with three colorimetric pH strips with a 0–14 pH range in 0.5 increments (Amazon, USA). The samples were placed on white paper next to the provided colorimetric key and photographed using a smartphone camera. The photos were analyzed to determine the color histogram of each pH strip using FIJI (ImageJ) and the color histograms compared to those of the key segments to determine the closest matching pH.

### Cell Culture

Human umbilical artery smooth muscle cells (SMCs; C-12500 Lot # 481Z014.1; PromoCell, Germany) were cultured in endothelial cell media supplemented with 5% fetal bovine serum (FBS), 100 units/mL penicillin, 100 μg/mL streptomycin, and 1% endothelial cell growth supplement (ScienCell, USA). Cells were cultured under sterile conditions at 37 °C and 5% CO_2_. Media was changed at least every 3 days and cells were passaged upon reaching ∼80% confluency.

### AlamarBlue Cytocompatibility Assay

SMCs were plated at a seeding density of 10,000 cells per well in a 96-well tissue culture-treated plate. Leached media was prepared as described above with a concentration of 400 mg hydrogel per mL of media. The cells were treated with varying concentrations of leached media: 100%, 75%, 50%, 25%, 10%, 1%, and 0% leached media with the balance being regular media. AlamarBlue readings were taken on days 0 (before treating the cells) and day 1. In a separate experiment, cells were treated with 100 μL of regular media and 100 μL of leached media (1%, 10%, 40%, or 80% w/v hydrogel in media) to maintain a 50:50 volume ratio between leached media and regular, complete media. AlamarBlue readings were taken on days 0 (before treating the cells), 1, 2, 3, 5, and 7. In both experiments, percent reduction in AlamarBlue was calculated according to manufacturer’s instructions. The percent reduction was normalized to the day 0 reading for each well. Media was changed every day to maintain a constant concentration of leachables in the media.

### Live/Dead Assay

SMCs were plated at a seeding density of 10,000 cells per well in a 48-well tissue culture-treated plate. Leached media was prepared as previously described at concentrations of 1% hydrogel in media and 80% hydrogel in media as the two extremes tested in the AlamarBlue assay. The cells were treated with 150 μL of regular media and 150 μL of leached media to maintain a 50:50 volume ratio between leached media and regular, complete media, resulting in exposure levels of 5 mg hydrogel per mL of media and 400 mg hydrogel per mL of media, respectively. Untreated cells, which were only exposed to regular, complete media, served as a control. Media was changed every other day to maintain a constant concentration of leachables in the media. Live/Dead assay was performed according to the manufacture’s protocol with 4 μM of Calcein AM (AAT Bioquest, USA) and 2 μM of Ethidium Homodimer II (Biotium, USA). The images were taken on days 1, 3, 5, and 7 following treatments. Images were obtained using Cytation 5 (Agilent, BioTek, USA) at 4× magnification with separate images obtained for green (live) and red (dead) channels. Images were merged using ImageJ.

### Liver Biopsy Punch Study

This study was conducted according to Texas A&M University IACUC Protocol #2024–0301. Briefly, Sprague–Dawley rats were anesthetized using isoflurane in oxygen gas and ensured to be in a surgical plane of anesthesia. The liver was exposed and a 6 mm cavity was created using a biopsy punch (Medline, USA). The cavity was observed to be bleeding freely, and the treatment administered immediately. Each rat received exactly one of the following treatments: no treatment, FloSeal, or PDA-gel/nSi hydrogel. Blood loss was measured using preweighed gauze placed underneath the liver. Clotting time was determined by three independent reviewers observing for lack of blood flow from video recordings of the surgery and averaged. After clotting was determined, the abdominal cavity was closed with wound clips (Braintree Scientific, USA) and the animal was monitored for 1 h. After 1 h, the abdominal cavity was opened to observe the injury site. The animal was euthanized and the liver was collected immediately postmortem. Two male rats and two female rats were used for each treatment group.

### Liver Biopsy Tissue Sections

Livers were excised immediately postmortem and submerged in 10% neutral buffered formalin (Avantor, USA) for at least 72 h and remained submerged until sectioning. Livers were washed three times in PBS to remove formalin, trimmed using a scalpel blade, embedded in Tissue-Tek O.C.T. Compound (Sakura Finetek, USA) and frozen at −80 °C overnight. Samples were sectioned at 20 μm thickness using a Cryostat Leica CM1950 (Leica, Germany) at −20 °C. Sections were placed on glass slides (Avantor, USA) and imaged at 1.6X and 8× magnification using a Zeiss SteREO microscope (Zeiss, Germany).

### Benchtop Uterine Flow Model

The model was constructed within a 250 mL glass beaker to physically confine the body of the model. A flow network was constructed from polypropylene tubing (Amazon, USA) with an internal diameter of 2.5 mm for the primary tubing and an internal diameter of 0.5 mm for the protruding flow network outlets. The primary tubing was molded into a spiral structure and secured using super glue. Nine precise piercings, evenly spaced by 2 mm, were made into each of 3 concentric arcs of the spiral structure using a 27G needle (Amazon, USA), creating 27 total perforations. At each perforation, a 1 cm × 0.5 mm tubing segment was inserted perpendicular to the lateral surface of the primary tubing to direct the flow upward to the surface of the model. These inserted segments were secured using clear nail polish (Amazon, USA). Thirty mL of PermaGel (Ballistic Dummy Lab, USA) was melted in an oven at 120 °C and poured into the 250 mL beaker. The flow network was submerged in the melted PermaGel such that approximately 5 mm of PermaGel was present between the opening ends of the inserted tubing segments and the surface of the PermaGel. A segment of porcine skin (Texas A&M Rosenthal Meat Center, USA) was applied to the surface of the model and adhered using super glue. The porcine skin provided a hydrophilic, cellular environment to evaluate the adhesion capability of the hydrogel. Perforations were made using a 27G needle to connect the surface of the porcine skin to the inserted tubing segments to allow flow to reach the surface of the model.

### Benchtop Uterine Flow Evaluation

The completed model was connected to a prefilled syringe of PBS for flow rate-pressure measurements. The syringe was placed in a custom 3D-printed stand and emptied at a controlled rate using a mechanical tester (MTest Quattro, ADMET, USA). Flow rates of 1 mL/min, 5 mL/min, 10 mL/min, and 15 mL/min were evaluated. Force measurements were recorded for approximately 30 s under each condition, and the average and maximum force values were determined. Average force was defined as the average force taken between 10 and 30 s of pumping to examine only the stabilized force.

The flow evaluation was also conducted with flow rates at every 1 mL/min between 5 mL/min and 10 mL/min with the manometer connected to a digital manometer (Amazon, USA) to determine the relationship between experimental pressure and flow rate. The maximum pressure was recorded once fluid was observed to be flowing freely on the surface of the model. This pressure/flow rate relationship was subsequently used to inform the flow rate used for the benchtop model testing to provide a quantitative comparison between the PDA-Gel/nSi hydrogel and FloSeal (described below).

### Benchtop Model Testing with Hydrogel

A syringe filled with red-dyed PBS was connected to the model flow network and placed in a custom stand to support the syringe in a mechanical tester (MTest Quattro, ADMET, USA). The syringe and mechanical tester delivered fluid through the model at a rate of 5 mL/min. The fluid was observed to flow freely through the perforations in the model. Afterward, a hydrogel sample was administered onto the surface of the model using a 16G blunt-tip needle (Amazon, USA) and a 1 mL syringe (Amazon, USA). The fluid was allowed to continue pumping for approximately 15 s, after which the model was tilted to observe for adhesion of the hydrogel to the surface. Four replicate experiments were conducted to observe consistency in the qualitative evaluation.

This same setup was conducted with a flow rate of 9 mL/min to achieve pressures above 80 mmHg according to results determined from the uterine flow evaluation pressure testing. Red-dyed PBS was pipetted onto the surface of the model prior to testing to create a wet environment. The sample (FloSeal or PDA-Gel) was then applied to the surface to cover the outlet holes. The pressure was monitored to determine the maximum pressure at which the sample began to leak and detach from the surface of the model. Three replicate experiments were conducted.

### Uterine Laceration Study

This study was conducted according to Texas A&M University IACUC Protocol #2024–0262. Briefly, pregnant, midterm (E14–15) Sprague–Dawley rats were anesthetized using isoflurane in oxygen gas and ensured to be in a surgical plane of anesthesia. The uterus was exposed and preweighed gauze was placed around and under the uterus. The uterus was lacerated using a sterile scalpel blade (Amazon, USA) and the fetuses and placentas were removed with forceps. Fetuses were euthanized via decapitation. The uterus was observed for bleeding. In the progesterone pretreatment group, 32 mg/kg progesterone (Henry Schein, USA) was administered intramuscularly in the hindlimb 10 min prior to beginning the laceration. In the PDA-gel/nSi hydrogel treatment group, the hydrogel was applied to the uterus using a syringe and a 14G blunt-tip needle. The uterus was left open to observe for contractions and stability of the hydrogel. After 30 min, the gauze was removed and weighed. The animal was monitored for a total of 1 h prior to euthanasia. Clotting time was not able to be measured due to the extremely low blood flow and inability to reliably identify the point at which blood was clotted. Three female rats were used for each treatment group.

### Swelling Ratio

PDA-gel/nSi hydrogel was placed into a preweighed 5 mL centrifuge tube and centrifuged at 2000*g* for 5 min to obtain a consistent surface area among replicates. Tubes and sample masses were recorded. Samples were incubated with deionized water, PBS, fresh citrated human plasma, or fresh citrated human whole blood. Citrated blood and plasma were used in order to avoid clotting in contact with the hydrogel and subsequently artificially inflated mass values. Plasma was obtained by centrifuging citrated blood at 1700*g* for 5 min and collecting the supernatant. Samples were incubated for 24 h at 37 °C. Afterward, the solution was removed and the mass of the tube and hydrogel was obtained. Swelling ratio was calculated as follows:

6 $$Q=\frac{m_{f}-m_{i}}{m_{i}}$$

where *Q* is the swelling ratio, *m*_f_ is the final mass of the hydrogel, and *m*_i_ is the initial mass of the hydrogel. Three hydrogel samples were utilized in each of three independent replicate experiments for a total of *N* = 9 swelling ratio calculations per solution. Unique blood donors were used for each of the three replicate experiments.

### Accelerated Degradation Study

Hydrogel samples were placed into preweighed microcentrifuge tubes and subsequently weighed to determine the mass of the hydrogel. Samples were centrifuged at 2000*g* for 5 min to obtain a consistent surface area across replicates. One mL of degradation solution (20% hydrogen peroxide (Avantor, USA), 0.1 N sodium hydroxide (Avantor, USA), or 5 U/mL collagenase type 4 (Worthington Biochemical, USA)) was gently pipetted on top of the sample. All samples were incubated at 37 °C. At specified time points, the samples were removed from the incubator and centrifuged at 500*g* for 10 min to collect nondegraded fragments and avoid aspirating nondegraded material. The degradation solution was pipetted out to carefully remove the solution without removing the hydrogel and the tube was weighed. Percent mass remaining was calculated as follows:

7 $$\mathrm{Mass Remaining}\;\left(\%\right)=\frac{m_{f}}{m_{i}}\times 100\%$$

where *m*_f_ is the wet mass of the hydrogel at the time point and *m*_i_ is the wet mass of the hydrogel initially. These masses were determined by weighing the hydrogel in its respective microcentrifuge tube and subtracting the weight of the centrifuge tube which was measured prior to adding the hydrogel. Time points up to 12 h each had their own samples. For time points at 24 h and beyond, the degradation solution was replaced and the sample was returned to the incubator. The degradation solution was replaced every 24 h in accordance with the time points and to maintain a constant exposure concentration of the degradation solutions.

### Dynamic Light Scattering

Nanosilicate was mixed with deionized water at a concentration of 0.1 mg/mL and allowed to exfoliate for 1 h. Exfoliated nanosilicate at a concentration of 2 mg/mL was combined with oxytocin (Astatech Inc, USA) at a concentration of 2 mg/mL to yield a final solution with 1 mg/mL nanosilicate and 1 mg/mL oxytocin. This mixture was allowed to rest for 1 h prior to testing. Dynamic light scattering was performed on a Malvern Zetasizer Nano ZS (Malvern Panalytical, UK) at 25 °C with deionized water as the solvent. Three repeat measurements were obtained for each sample.

### Zeta Potential

Nanosilicate was mixed with deionized water at a concentration of 1 mg/mL and allowed to rest for 1 h prior to testing. A nanosilicate-oxytocin mixture was obtained by combining equal proportions of 1 mg/mL nanosilicate and 1 mg/mL oxytocin. Oxytocin was dissolved in deionized water at 1 mg/mL. All solutions were allowed to rest for 1 h prior to testing. Zeta potential was measured using a Malvern Zetasizer Nano ZS (Malvern Panalytical, UK) at 20 °C with deionized water as the solvent. Three repeat measurements were obtained for each sample.

### Statistical Analysis

Statistical analysis was performed using GraphPad Prism 9 (GraphPad, USA). Specific statistical tests, sample sizes, and any deviations are noted in the figure captions of the corresponding data. Multiple biological replicates are noted when used.

## Supplementary Material









## Data Availability

All raw data and analysis files can be found at the following Zenodo repositories: 10.5281/zenodo.20670318 (for Figures 1–3 and associated Supporting Information Figures) and 10.5281/zenodo.20671537 (for Figure 4 and associated Supporting Information Figures).

## Notes

Detailed materials and methods; Figures S1–S18 include materials characterization, in vitro assays, rheological and injectability testing, sterilization and cytocompatibility studies, swelling and degradation analyses, oxytocin loading studies, and preclinical evaluation in a rat uterine laceration model (PDF)
Video S1: No-treatment uterine laceration (MP4)
Video S2: Progesterone pretreatment uterine laceration (MP4)
Video S3: PDA-gel/nSi hydrogel treatment uterine laceration (MP4)
