# Supplementary material for: Adhesive and Hemostatic Hydrogel for the Management of Postpartum Hemorrhage
Source: ACS Appl Mater Interfaces. 2026 Jun 22;18(25):34835–56. doi: 10.1021/acsami.6c05034 (PMC13339009; doi:10.1021/acsami.6c05034)
Supplement: Supplementary file 1 [file am6c05034_si_001.pdf]

Supporting Information

Adhesive and Hemostatic Hydrogel for Management of Postpartum Hemorrhage

Sarah E. Miller[1], Prasenjeet Ingole[2], Anya Stolyarova[2], Dylan Po[3], Tripp D. Moss[4], Jasmine Sureka[1], Saptarshi Biswas[1], Ryan Davis, Jr.[1], Akhilesh K. Gaharwar[1,4,5,6*]

[1]Department of Biomedical Engineering, College of Engineering, Texas A&M University, College Station, TX, 77843, USA

[2]Department of Biology, College of Arts and Sciences, Texas A&M University, College Station, TX, 77843, USA

[3]School of Public Health, Texas A&M University, College Station, TX, 77843, USA

[4]Department of Materials Science and Engineering, Texas A&M University, College Station, TX, 77843, USA

[5]Interdisciplinary Program in Genetics, Texas A&M University, College Station, TX, 77843, USA

[6]Center for Remote Health Technologies and Systems, Texas A&M University, College Station, TX, 77843, USA

[*]Corresponding author; gaharwar@tamu.edu

A two-step polydopamine-gelatin synthesis resulted in high catechol content.

We began by conjugating polydopamine onto a gelatin backbone using a one-pot synthesis method with 1-Ethyl-3-(3-dimethylaminopropyl) carbodiimide (EDC) and N-Hydroxysuccinimide (NHS) chemistry (**Fig S1A**) based on previously published literature[1-6]. Although this synthesis was intended to graft dopamine directly onto the gelatin backbone, the resulting polymer displayed a brown coloring, indicating that polydopamine had formed, rather than unpolymerized dopamine[7]. This observation is consistent with previous literature indicating that self-polymerization of dopamine is common when using carbodiimide crosslinker chemistry[8], as was used in this study. With each synthesis, we observed clear water following dialysis of the polymer to confirm long polymer chains with grafted polydopamine rather than inclusion of nanoparticles. Initial exploration with the resulting polymer yielded only weak adhesion, so we explored the impact of the reagent ratios on the presence of catechol groups in the final polymer (**Fig S1B**). Various reaction modifications were performed and the catechol content of the resulting polymer was quantitatively assessed using Arnow's Method[9]. While increasing the relative presence of NHS simultaneously or increasing the presence of dopamine in the reaction did yield notable improvement in the dopamine content of the resulting polymer, these increases were not proportional to the increase in reagents. Thus, we sought to alter other aspects of the synthesis to improve the catechol content of the final polymer.

The speed of reagent addition was one such consideration. Typically, a wait period occurs between the addition of EDC/NHS reagents and the addition of dopamine to maximize the full priming of the carboxyl groups and thus the ability of the carboxyl groups to accept the amine group from dopamine[1-3]. We compared the dopamine content of polymers synthesized with and without this waiting period and observed that the waiting period did result in higher dopamine conjugation (**Fig S1C**). For the next consideration, the typical buffer solution, 2-(N-morpholino) ethanesulfonic acid (MES) held at pH 4.5[10], was replaced with phosphate buffered saline (PBS) at pH 7.4. This alteration yielded significantly higher catechol content in the resulting polymer (**Fig S1D**), despite no change in the reagent ratios. Previous literature indicates that dopamine conjugation occurs spontaneously in alkaline environments under the presence of oxygen[7]. However, EDC/NHS chemistry is known to be more efficient in an acidic solution[10] and phosphate groups may also be reacted into the final product[10]. Therefore, we next considered a reaction conducted in MES buffer to obtain an initially acidic environment and subsequently adjusted the pH to achieve a more basic environment for dopamine conjugation. We conducted a series of syntheses in MES buffer with constant reagents and adjusted the pH immediately prior to dopamine addition. The dopamine content in the resulting polymers remained relatively consistent across pH ranging from 6 to 10 but increased significantly when the reaction solution was adjusted to pH 12 (**Fig S1E**). In parallel, polydopamine synthesis is known to occur in notably alkaline environments containing ethanol and basic agents[11] which we observed to result in a pH between 12-13, so we hypothesize that the increased dopamine content was due to increased polydopamine polymerization into long chains, which were subsequently retained in the dialysis tubing, rather than conjugation onto the gelatin backbone. Thus, we selected a pH of 8 to perform subsequent syntheses in an attempt to achieve the increased conjugation seen in the PBS-buffered synthesis without altering the final polymer composition.

The selection of pH 8 is also supported by the process conditions for solution oxidation of dopamine to produce polydopamine[7] and for reaction of the dopamine amine group with the NHS-ester[12, 13]. Some prior literature indicates that polydopamine-conjugated materials can demonstrate improved adhesion over dopamine-conjugated materials due to increased ability to engage in pi-pi interactions with nonpolar biological tissues[14, 15]. Polydopamine can be synthesized using several processes, including solution

oxidation, enzymatic-driven oxidation, and electropolymerization[7]. Solution oxidation, in which the dopamine raw material is reacted in an oxidative, alkaline environment[11] and undergoes auto-polymerization, is the most commonly used due to ease of use and simple reagent requirements[7]. In particular, the reaction can be left exposed to the atmosphere and atmospheric oxygen serves as an oxidant for the reaction[11]. Previous work by Wu et al noted that this technique also requires an initial dopamine concentration above 2 mg/mL[7], so maintaining a concentration below this may assist in balancing polydopamine nanoparticle formation with conjugation to gelatin. Wu et al also described in their work how adjusting synthesis conditions such as increasing the pH and sodium hydroxide (NaOH) content or the temperature results in faster reaction kinetics and increased nucleation sites for polydopamine formation, resulting in smaller polydopamine cores[11]. Additionally, previous literature indicating that dopamine reaction with the NHS-ester occurs most effectively at pH 7-8[12, 13], thus enabling the formation of a stable polymer rather than retaining free dopamine present in the hydrogel system. Following the observations noted above, we explored the impact of preparing polydopamine separately prior to conjugation onto gelatin.

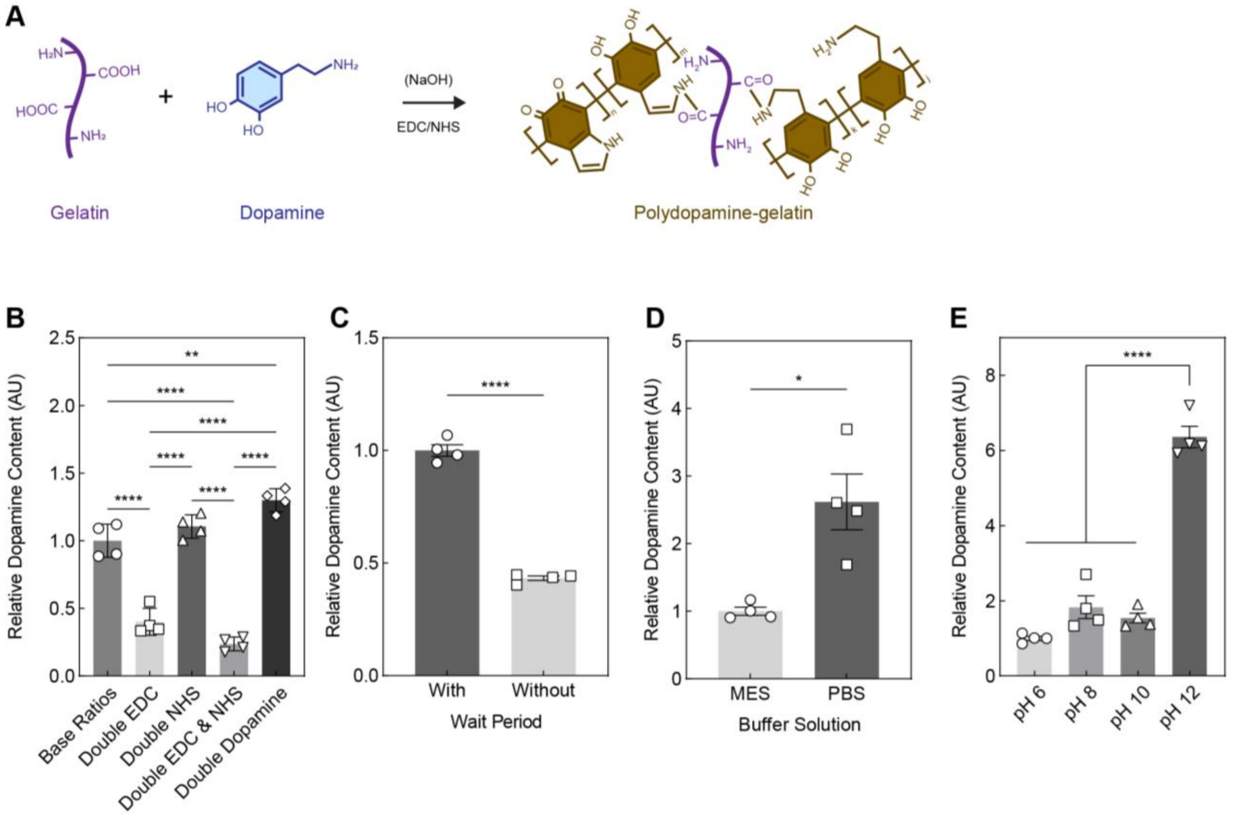

Figure S1. Polydopamine-gelatin synthesis optimization.
A) Schematic showing one-pot synthesis scheme to produce polydopamine-conjugated gelatin from gelatin and dopamine.
*B) Relative dopamine content among polymers synthesized with varying reagent ratios. N = 4; data shown is mean ± standard deviation. Ordinary one-way ANOVA with Tukey's multiple comparisons test. *p<0.05, **p<0.01, ***p<0.001, ****p<0.0001. Data is normalized to the mean of leftmost dataset to facilitate comparisons. AU: arbitrary units.*
*C) Relative dopamine content among polymers synthesized with or without a 20-minute wait period between EDC/NHS addition and dopamine addition. N = 4; data shown is mean ± standard error of the mean. Welch's t-test. *p<0.05, **p<0.01, ***p<0.001, ****p<0.0001. Data is normalized to the mean of leftmost dataset to facilitate comparisons. AU: arbitrary units.*

D) Relative dopamine content among polymers synthesized in MES buffer or PBS buffer. N = 4; data shown is mean ± standard error of the mean. Welch's t-test. *p<0.05, **p<0.01, ***p<0.001, ****p<0.0001. Data is normalized to mean the of leftmost dataset to facilitate comparisons. AU: arbitrary units.

E) Relative dopamine content of polymers synthesized with varying pH adjustments prior to adding dopamine. N = 4; data shown is mean ± standard error of the mean. Ordinary one-way ANOVA with Tukey's multiple comparisons test. *p<0.05, **p<0.01, ***p<0.001, ****p<0.0001. Data is normalized to the mean of leftmost dataset to facilitate comparisons. AU: arbitrary units.

We performed three distinct syntheses to identify the optimal method for preparing polydopamine-gelatin (PDA-gelatin) (**Fig S2A**): first, with gelatin and dopamine reacted in a one-pot synthesis with spontaneously polymerized polydopamine as in the previously described syntheses ("PDA-Gelatin V0"), second, with less polydopamine pre-reacted and a subsequent reaction to gelatin to determine if equivalence could be obtained with less raw materials ("PDA-Gelatin V1"), and third, with equivalent polydopamine pre-reacted and a subsequent reaction to gelatin ("PDA-Gelatin V2"). Quantitative evaluation using Arnow's Method revealed that while polydopamine pre-reaction was able to achieve similar levels of dopamine content with a lower initial mass of dopamine compared to the one-pot synthesis, a significantly higher catechol content was present in PDA-Gelatin V2 polymers (**Fig S2B**). The PDA-Gelatin V2 polymers exhibited approximately 253.20 ± 15.90 ug catechol per mg of polymer, or ~25% catechol in the hydrogel system, representing a considerable increase over previous systems which is primarily attributed to the pH modulation throughout the reaction[3, 14]. Discussion of NMR and FTIR results appears in the main text. Based on these results, we hypothesized that PDA-gelatin V2 would present the greatest wet tissue adhesion.

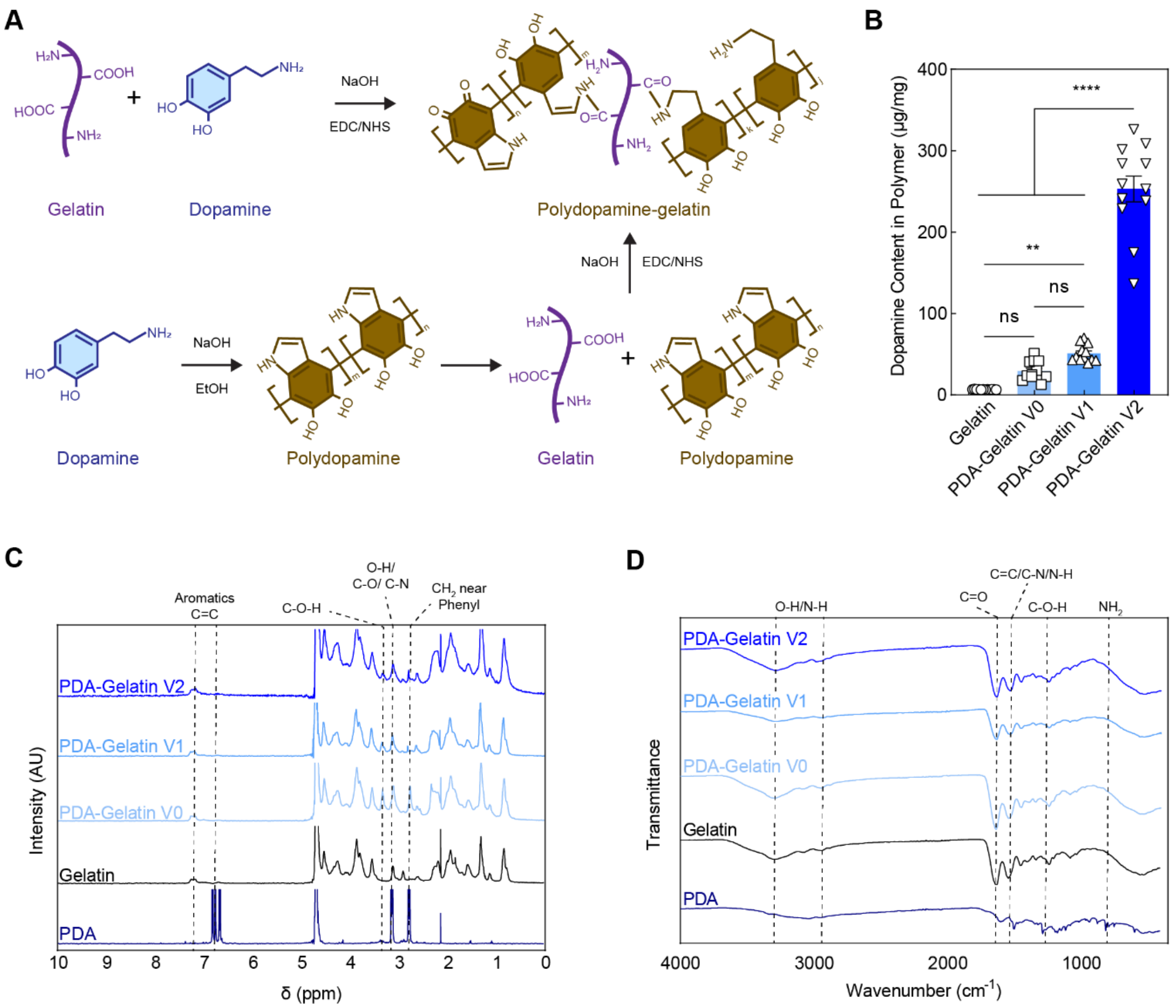

Figure S2. Polydopamine-gelatin synthesis evaluation.

A) Schematic showing one-pot (top; horizontal) synthesis schematic and two-pot (bottom; horizontal into vertical) synthesis schematic.

*B) Arnow's method analysis of dopamine content in PDA-Gelatin V0 (one pot), PDA-Gelatin V1 (polydopamine pre-reacted, less reagent), and PDA-Gelatin V2 (polydopamine pre-reacted, standard reagent amount). Gelatin serves as a control. N = 4 samples from each of three synthesis replicates (N = 12 total); data shown is mean ± standard error of the mean. Ordinary one-way ANOVA with Tukey's multiple comparisons test. *p<0.05, **p<0.01, ***p<0.001, ****p<0.0001.*

C) NMR analysis of PDA-Gelatin V0, PDA-Gelatin V1, and PDA-Gelatin V2. NMR spectra are shown in comparison to unmodified gelatin and PDA alone to highlight new bond formation present in the conjugated polymers. Representative spectra are shown.

D) FTIR analysis of PDA-Gelatin V0, PDA-Gelatin V1, and PDA-Gelatin V2. FTIR spectra are shown in comparison to unmodified gelatin and PDA alone to highlight new bond formation present in the conjugated polymers. Representative spectra are shown.

We first examined the wet-tissue adhesion capability using a lap-shear style test with porcine skin soaked in phosphate buffered saline (PBS). Although burst strength is more applicable to our study due to the desire to seal bleeding blood vessels, purely liquid samples cannot be effectively evaluated in this setup and will

instead fall into the associated tubing without sealing. A 10% solution of each polymer was prepared in deionized water and allowed to sit at room temperature prior to application onto the pig skin. The weight held by each sample was converted to force and normalized against the mass of the polymer solution applied. This was repeated for 20% solutions of each polymer. In both concentrations, PDA-Gelatin V2 demonstrated the strongest improvement compared to gelatin alone (**Fig S3A-B**). At the 10% concentration, PDA-Gelatin V2 also demonstrated increased adhesion over PDA-Gelatin V0. Despite lacking statistical significance in comparison to other groups, PDA-Gelatin V2 (hereafter referred to as “PDA-gelatin”) presented numerically increased adhesion capability in both the 10% and 20% concentrations. Therefore, we elected to continue the study with only this formulation.

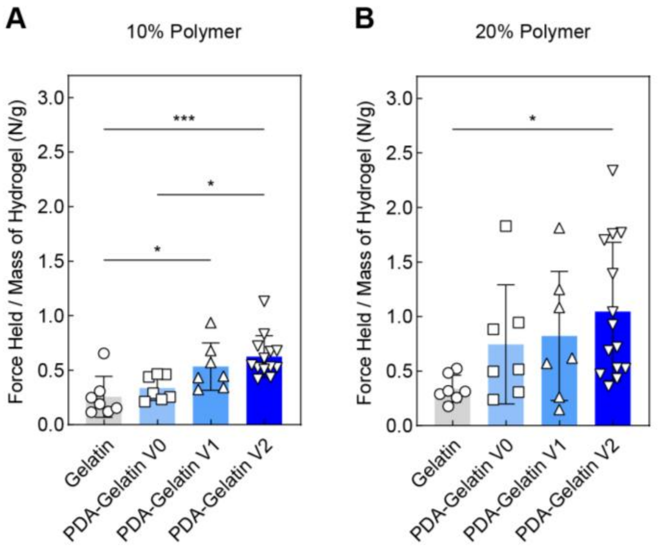

Figure S3. Polymer composition selection.
*A) Lap-shear style adhesion testing of 10% solutions of PDA-Gelatin V0, PDA-Gelatin V1, and PDA-Gelatin V2. Gelatin served as a control. N = 7 for gelatin, PDA-Gelatin V0, and PDA-Gelatin V1; N = 13 for PDA-Gelatin V2; data is mean ± standard deviation. Mass held is converted to force against gravity and normalized to the mass of hydrogel applied. Ordinary one-way ANOVA with Tukey’s multiple comparisons test. *p<0.05, **p<0.01, ***p<0.001, ****p<0.0001.*
*B) Lap-shear style adhesion testing of 20% solutions of PDA-Gelatin V0, PDA-Gelatin V1, and PDA-Gelatin V2. Gelatin served as a control. N = 7 for gelatin, PDA-Gelatin V0, and PDA-Gelatin V1; N = 14 for PDA-Gelatin V2; data is mean ± standard deviation. Mass held is converted to force against gravity and normalized to the mass of hydrogel applied. Ordinary one-way ANOVA with Tukey’s multiple comparisons test. *p<0.05, **p<0.01, ***p<0.001, ****p<0.0001.*

Hemostatic ability and adhesive capability were balanced to achieve a multifunctional, modified hydrogel.

After determining the polymer composition to be used in fabricating the hydrogel, we then examined the hemostatic ability with the intent of optimizing the final hydrogel composition to achieve both adhesion and hemostasis. We began by conducting a clotting time inversion test across a concentration sweep for PDA-gelatin. Dopamine has been previously shown to initiate clotting, with concentrations between 40 and 400 µg/mL promoting platelet aggregation[16]. Dopamine has also been utilized in a number of novel hemostatic materials, ranging from adhesive patches[14] to injectable biomaterials[12] to sponges and foams[17] with a variety of applications.

Consistent with this literature, we observed that solutions of polydopamine-gelatin reduced the clotting time compared to empty tubes (negative control) (**Fig S4A**) in an in vitro clotting time inversion test. However, increasing the dopamine content did not result in faster clotting; rather, only a weakly inverse trend was observed. Samples of 10% PDA-gelatin resulted in a clotting time reduction of 15.96 ± 16.04% from negative control, indicating highly variable results which were attributed to the highly liquid form of this sample. A similar result was observed for 20% PDA-gelatin and 40% PDA-gelatin, which presented clotting time reductions of 12.40 ± 5.31% and 13.84 ± 7.98%, respectively. No statistical significance was noted between the three concentrations. We noted that samples of higher concentrations, namely 40% PDA-gelatin, were difficult to mix homogeneously and frequently resulted in clumping within the sample tube. Based on these results and observations, we selected 10% and 20% PDA-gelatin for further evaluation in terms of hemostatic ability.

We then conducted the clotting time inversion test for hydrogels composed of either 10% or 20% PDA-gelatin with nanosilicate concentrations ranging from 0 to 10% (**Fig S4B**). Overall, increasing the nanosilicate concentration resulted in increased reduction in clotting time. This result is consistent with previous studies indicating that nanosilicates are able to accelerate clotting[18-20]. Interestingly, we observed a greater influence of nanosilicate in the 10% PDA-gelatin samples compared to the 20% PDA-gelatin samples. We hypothesized that this influence is due to the overall decreased mass percentage and concurrent decrease in physical crosslinks, which enabled better blood infiltration and allowed for greater interaction with the hydrogel. Finally, we examined these clotting time results in tandem with adhesive ability. We conducted additional lap shear-style testing for hydrogels composed of varying PDA-gelatin (10% or 20%) and varying nanosilicate (0, 2, 4, 6, 8, or 10%) (**Fig S4C**). Overall, we observed very little variation in adhesive ability as a result of changing nanosilicate concentration. Only one hydrogel composition, 20% PDA-gelatin 4% nanosilicate, demonstrated any significant increase in adhesive ability over any other group; however, this increase was only in comparison to 10% PDA-gelatin without nanosilicate and was not present in comparison to any of the other nanosilicate-containing formulations. When comparing the hydrogel compositions in terms of both adhesive ability and hemostatic ability (**Fig S4D**), we identified the hydrogel composition of 10% PDA-gelatin 10% nanosilicate as the optimization point between adhesive ability and hemostatic ability. Furthermore, although this composition did not present the highest numerical adhesive ability, it was not significantly different compared to any of the other compositions, and this composition did present the greatest reduction in clotting time with statistical significance compared to other groups. Based on these results, we selected this composition (10% PDA-gelatin 10% nanosilicate) for further evaluation as a potential treatment for postpartum hemorrhage.

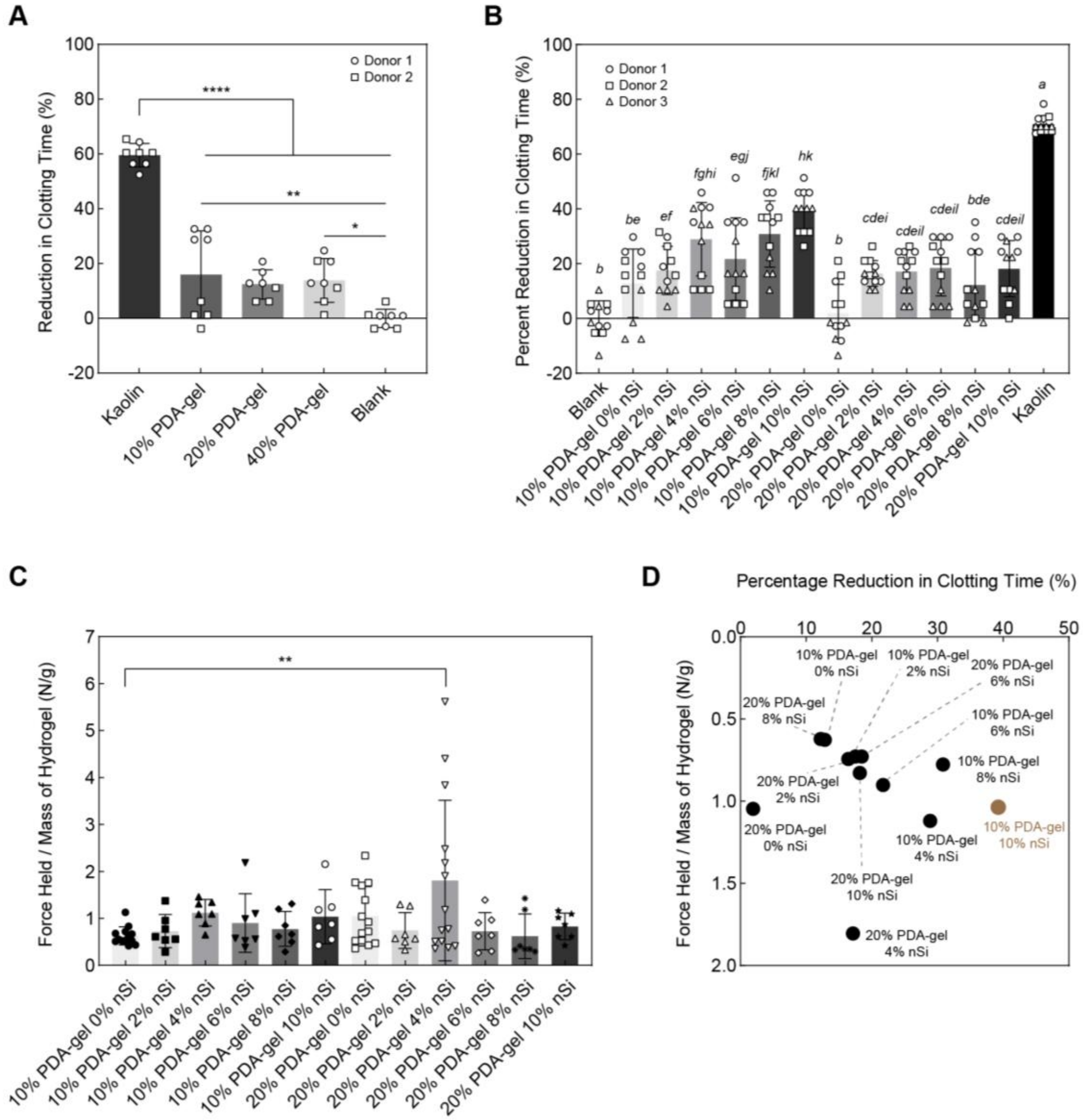

Figure S4. Hydrogel composition optimization for clotting.

*A) Clotting time inversion test for varying PDA-gelatin concentrations. Kaolin served as a positive control; empty tubes ("blank") served as a negative control. N = 7 across 2 unique donors for 20% PDA-gelatin due to damaged sample (N = 3-4 each donor); all other groups have N = 8 across 2 unique donors (N = 4 each donor). Data is mean ± standard deviation. Ordinary one-way ANOVA with Tukey's multiple comparisons test. *p<0.05, **p<0.01, ***p<0.001, ****p<0.0001.*

B) Clotting time inversion test for hydrogels composed of 10% PDA-gelatin or 20% PDA-gelatin with varying nanosilicate concentrations. Kaolin served as a positive control; empty tubes ("blank") served as a negative control. N = 11 across 3 unique donors (N = 3 -4 each donor) for 10% PDA-gelatin 2% nSi group due to damaged sample; all other groups have N = 12 across 3 unique donors (N = 4 each donor). Data is mean ± standard deviation. Ordinary one-way ANOVA with Tukey's multiple comparisons test. Letter in common shown above the bar indicates no statistically significant difference; otherwise, a statistically significant difference of at least p<0.05 is indicated.

C) Lap-shear style adhesion testing of hydrogels containing either 10% or 20% PDA-gelatin and nanosilicate concentrations ranging from 0% to 10%. N = 13 for 10% PDA-gelatin 0% nSi, N = 14 for 20% PDA-gelatin 0% nSi, N = 14 for 20% PDA-gelatin 4% nSi, N = 7 for all other groups. Data shown is mean ± standard deviation. Mass held is converted to force against gravity and normalized to the mass of hydrogel applied. Ordinary one-way ANOVA with Tukey's multiple

*comparisons test. *p<0.05, **p<0.01, ***p<0.001, ****p<0.0001. Note that data for 10% PDA-gelatin 0% nSi and for 20% PDA-gelatin 0% nSi are reproduced from that shown for PDA-gelatin V2 in Figures S3A and S3B, respectively.*
D) Comparison of clotting ability vs adhesive ability. Points shown are at the average clotting time (individual data points shown in Figure S4B) and average force held per mass of hydrogel (individual data points shown in Figure S4C). Error bars are not shown for visual clarity.

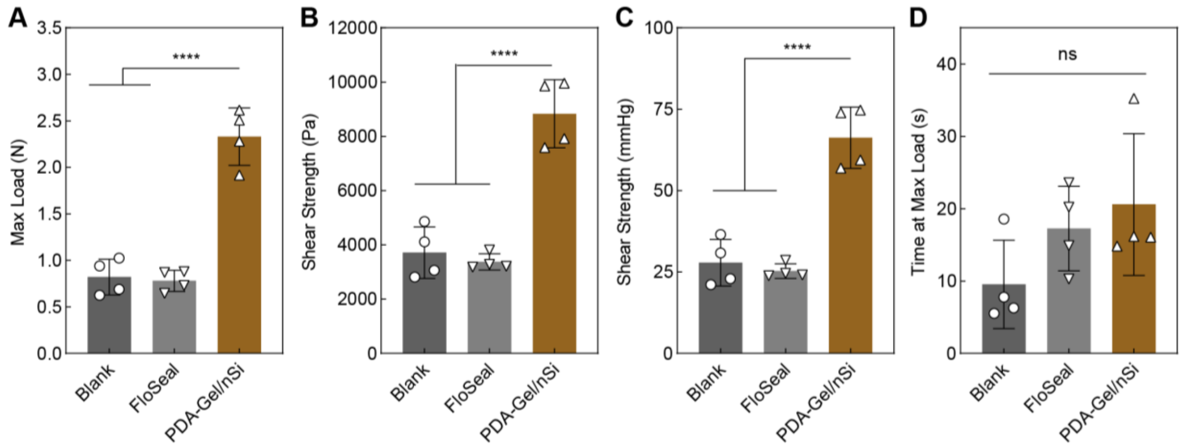

Figure S5. Lap shear testing quantitative results.
*A) Maximum loading force sustained by each sample: PDA-gelatin/nSi hydrogel, clinical control FloSeal, and skin samples without additional bonding agent. N = 4; data shown is mean ± standard deviation. Ordinary one-way ANOVA with Tukey's multiple comparisons test. *p<0.05, **p<0.01, ***p<0.001, ****p<0.0001.*
*B) Shear strength in Pascals determined by dividing the maximum loading force by the bond surface area of the specimen. N = 4; data shown is mean ± standard deviation. Ordinary one-way ANOVA with Tukey's multiple comparisons test. *p<0.05, **p<0.01, ***p<0.001, ****p<0.0001.*
*C) Shear strength in millimeters of mercury to facilitate comparison with burst pressure. Data is converted from that in Figure S5B. N = 4; data shown is mean ± standard deviation. Ordinary one-way ANOVA with Tukey's multiple comparisons test. *p<0.05, **p<0.01, ***p<0.001, ****p<0.0001.*
*D) Time at maximum loading force. The time elapsed since test start at the time of the maximum loading force is compared for each sample. N = 4; data shown is mean ± standard deviation. Ordinary one-way ANOVA with Tukey's multiple comparisons test. *p<0.05, **p<0.01, ***p<0.001, ****p<0.0001.*

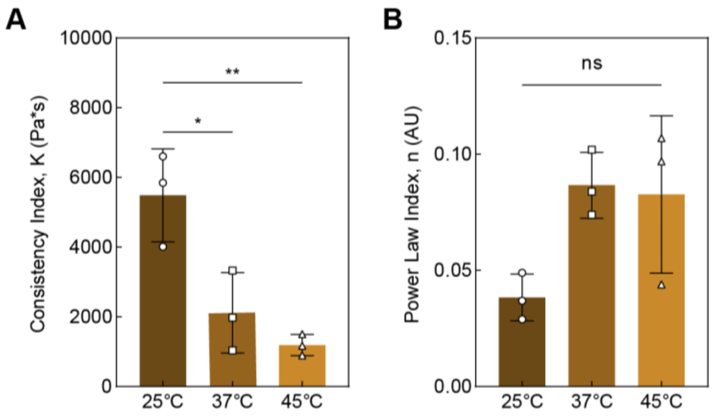

Figure S6. Shear rate sweep power law values.

*A) Consistency index, K, derived from a power-law fit to the viscosity-vs-shear rate curve, indicating the theoretical viscosity of an equivalent Newtonian fluid. N = 3; data shown is mean ± standard deviation. Ordinary one-way ANOVA with Tukey's multiple comparisons test. *p<0.05, **p<0.01, ***p<0.001, ****p<0.0001.*

*B) Power law index, n, derived from a power-law fit to the viscosity-vs-shear rate curve, indicating the shear-thinning behavior of the viscoelastic fluid. N = 3; data data shown is mean ± standard deviation. Ordinary one-way ANOVA with Tukey's multiple comparisons test. *p<0.05, **p<0.01, ***p<0.001, ****p<0.0001.*

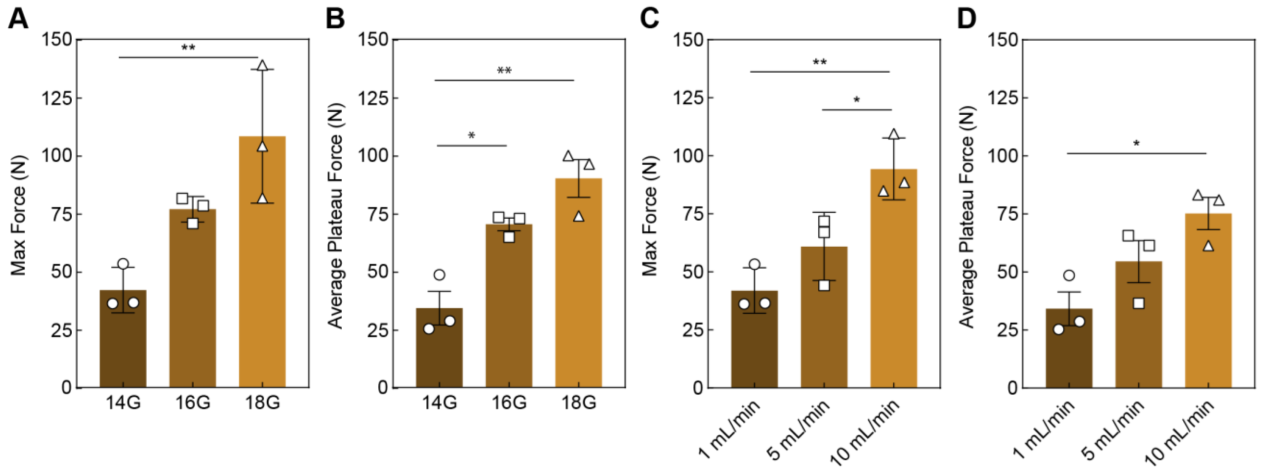

Figure S7. Injection force testing comparisons.

*A) Maximum force generated while extruding PDA-gel/nSi hydrogel from a 1 mL syringe with various blunt tip needles (14G, 16G, and 18G) at a flow rate of 1 mL/min. Max force is determined from data up to 0.95 mL extruded to avoid increases in force resulting from the syringe plunger impacting the end of the syringe barrel. N = 3; data shown is mean ± standard deviation. Ordinary one-way ANOVA with Tukey's multiple comparisons test. *p<0.05, **p<0.01, ***p<0.001, ****p<0.0001.*

*B) Average plateau force generated while extruding PDA-gel/nSi hydrogel from a 1 mL syringe with various blunt tip needles (14G, 16G, and 18G) at a flow rate of 1 mL/min. Average plateau force is determined from data between 0.25 mL and 0.95 mL extruded to determine the stabilized force. N = 3; data shown is mean ± standard error of the mean. Ordinary one-way ANOVA with Tukey's multiple comparisons test. *p<0.05, **p<0.01, ***p<0.001, ****p<0.0001.*

*C) Maximum force generated while extruding PDA-gel/nSi hydrogel from a 1 mL syringe with a 14G blunt tip needles at various flow rates (1, 5, and 10 mL/min). Max force is determined from data up to 0.95 mL extruded to avoid increases in force resulting from the syringe plunger impacting the end of the syringe barrel. N = 3; data shown is mean ± standard deviation. Ordinary one-way ANOVA with Tukey's multiple comparisons test. *p<0.05, **p<0.01, ***p<0.001, ****p<0.0001.*

*D) Average plateau force generated while extruding PDA-gel/nSi hydrogel from a 1 mL syringe with a 14G blunt tip needles at various flow rates (1, 5, and 10 mL/min). Average plateau force is determined from data between 0.25 mL and 0.95 mL extruded to determine the stabilized force. N = 3; data shown is mean ± standard error of the mean. Ordinary one-way ANOVA with Tukey's multiple comparisons test. *p<0.05, **p<0.01, ***p<0.001, ****p<0.0001.*
Note: data for 14G, 1 mL/min test condition appears in both Fig S8A-B and in Fig S8C-D.

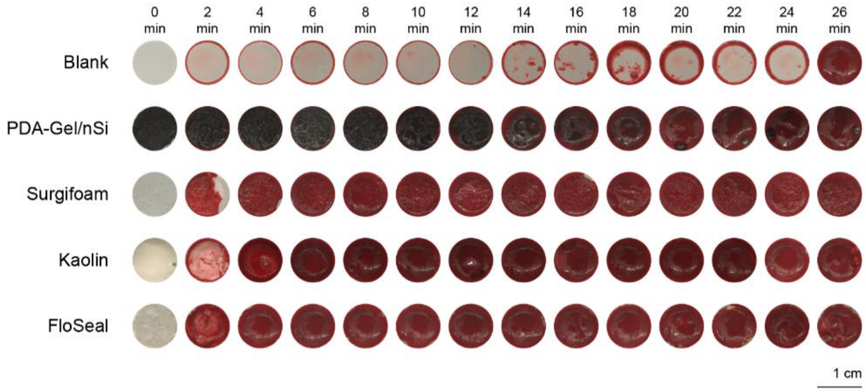

Figure S8. Bovine blood clotting well plate (48-well size). *The static clotting assay (as seen in **Figure 2B**) was conducted again, this time in a 48-well plate. Samples were placed into the wells of a 48-well plate and incubated with recalcified whole bovine blood for specified time points. At each time point, unclotted blood was carefully aspirated and the well was photographed. FloSeal was observed to achieve clotting fastest, at 4 minutes. Kaolin and Surgifoam each achieved clotting within 6 minutes. PDA-gel/nSi achieved clotting within 14 minutes. Blank wells without any treatment required 26 minutes to achieve clotting. The increased stratification of the clotting times due to the larger volume of blood allows for a better understanding of the trends in clotting among the sample types.*

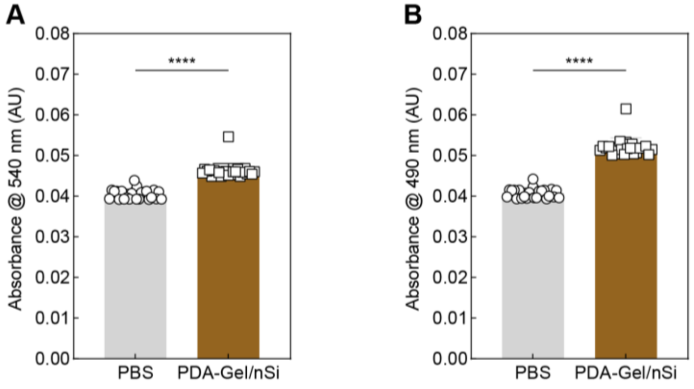

Figure S9. Absorbance interference due to PDA-gelatin.
*A) Absorbance of PBS sample vs PDA-gel/nSi sample read at 540 nm after undergoing incubation consistent with red blood cell adhesion, platelet adhesion, and hemolysis assays. N = 24 across 6 samples (N = 4 each sample); data shown is mean ± standard error of the mean. Unpaired t-test. *p<0.05, **p<0.01, ***p<0.001, ****p<0.0001.*
*B) Absorbance of PBS sample vs PDA-gel/nSi sample read at 490 nm after undergoing incubation consistent with red blood cell adhesion, platelet adhesion, and hemolysis assays. N = 24 across 6 samples (N = 4 each sample); data shown is mean ± standard error of the mean. Unpaired t-test. *p<0.05, **p<0.01, ***p<0.001, ****p<0.0001.*

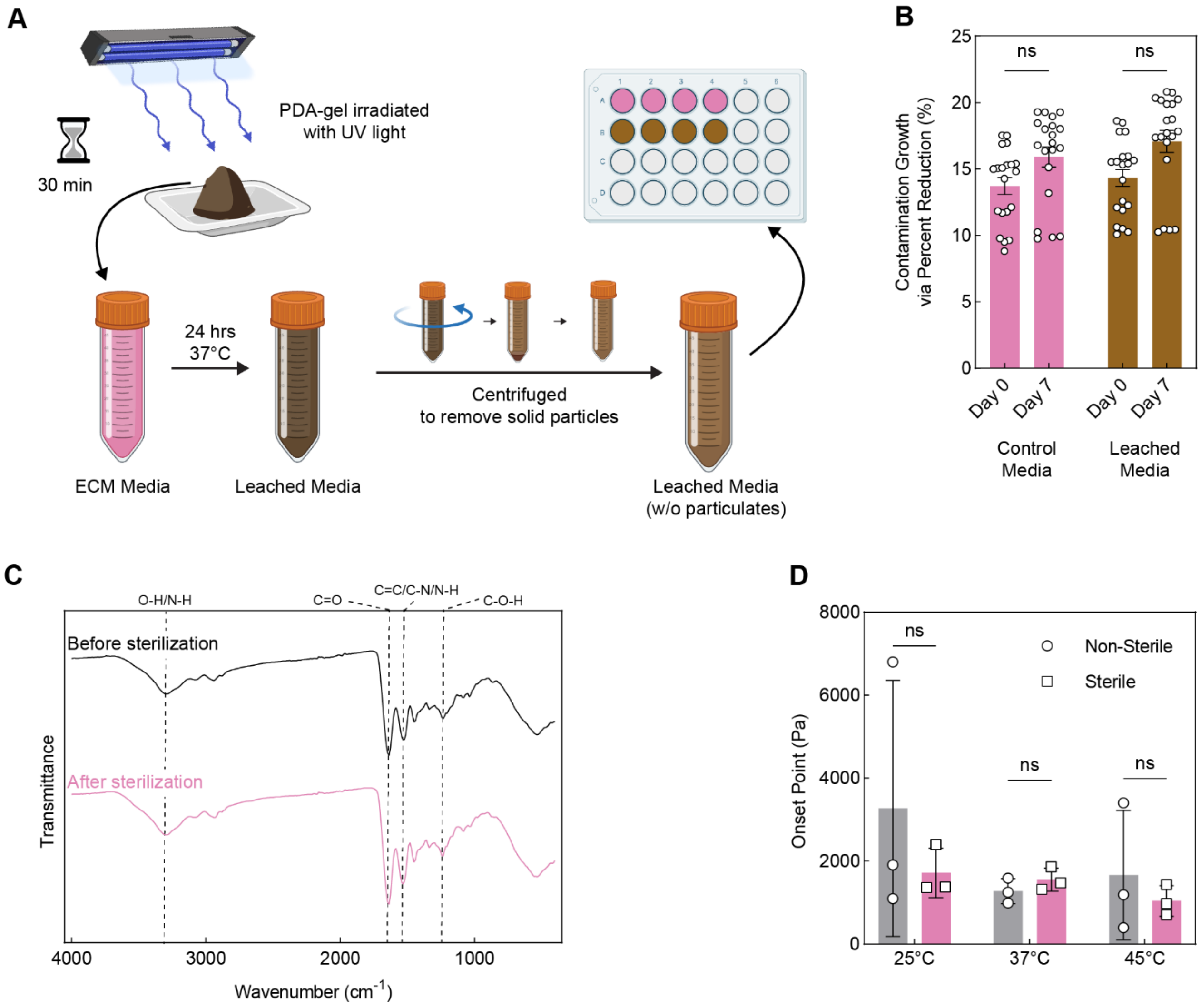

Figure S10. Sterilization procedure validation.

A) Schematic of sterilization procedure and validation via Alamar Blue in comparison to sterile, control media. Schematic was made in part using Biorender.com.

*B) Identification of contamination/non-sterile material via percent reduction of Alamar Blue dye. Complete endothelial cell media serves as a sterile control. N = 20 across five sterilized polymer samples (N = 4 each polymer sample); data is mean ± standard error of the mean. Two-way ANOVA with Bonferroni's multiple comparisons test. *p<0.05, **p<0.01, ***p<0.001, ****p<0.0001.*

C) FTIR spectra of polymer before and after sterilization. Representative spectra are shown.

*D) Onset point of storage modulus from stress sweeps at 25°C, 37°C, and 45°C for both non-sterile and sterilized hydrogels. N = 3; data shown is mean ± standard deviation. Two-way ANOVA with Bonferroni's multiple comparisons test. *p<0.05, **p<0.01, ***p<0.001, ****p<0.0001.*

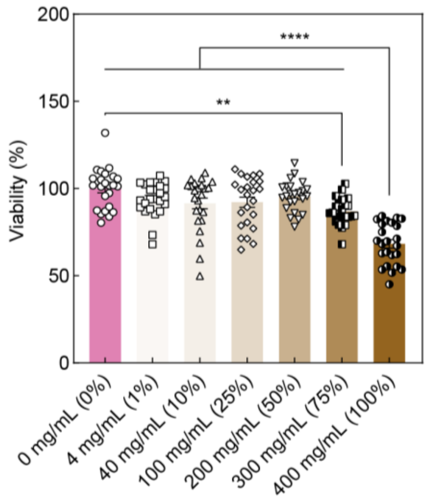

Figure S11. Cellular viability with varying dilution of leached media.
*Cellular viability expressed as a percent reduction of Alamar Blue after 1 day of exposure to leached media at varying concentrations. N = 24 across six unique hydrogel leached medias (N = 4 each hydrogel); data shown is mean ± standard error of the mean. One-way ANOVA with Tukey's multiple comparisons test. *p<0.05, **p<0.01, ***p<0.001, ****p<0.0001.*

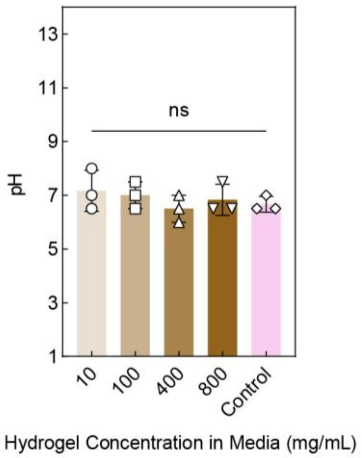

Figure S12. Impact of hydrogel on cell culture media pH.
*pH measurements of cell culture media samples exposed to 10, 100, 400, and 800 mg hydrogel per mL of media. Regular, unexposed complete media serves as a control. N = 3; data shown is mean ± standard deviation. One-way ANOVA with Tukey's multiple comparisons test. *p<0.05, **p<0.01, ***p<0.001, ****p<0.0001.*

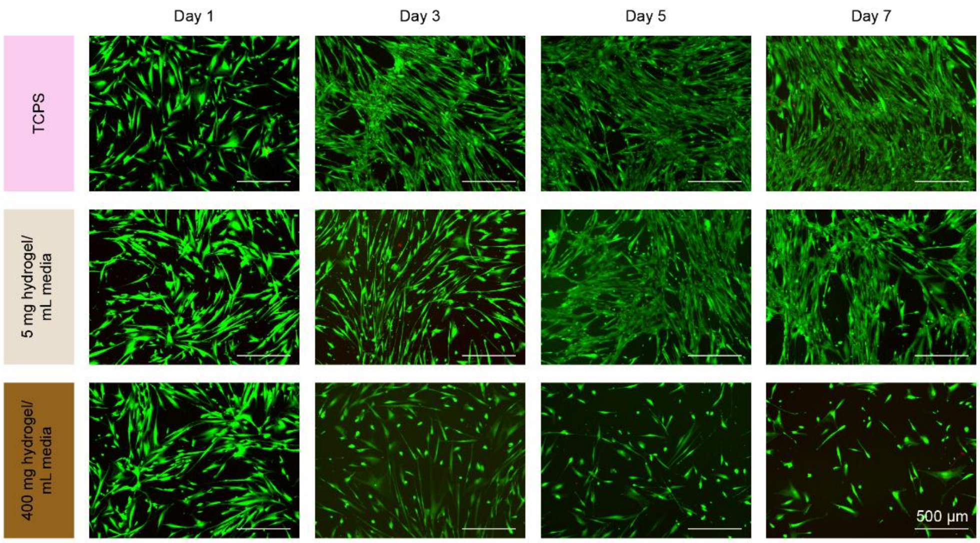

Figure S13. Live/dead assay.

SMC cells were indirectly exposed to leached media containing 5 or 400 mg of hydrogel per mL of media. Representative composite images with live (green) and dead (red) channels are shown for days 1, 3, 5, and 7 at 4X magnification. Tissue culture polystyrene (TCPS) served as a control. Original images are available in Zenodo repository.

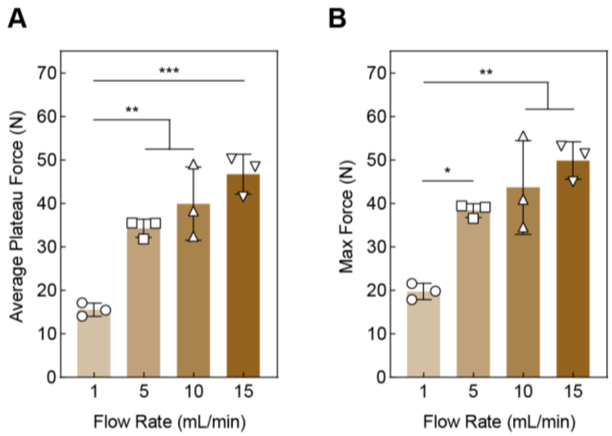

Figure S14. Benchtop model parameters and flow testing.

*A) Average force generated by pumping fluid at various flow rates through the model. Average plateau force is defined as the average force taken after 10 seconds of pumping to examine only the stabilized force. N= 3; data shown is mean ± standard deviation. Ordinary one-way ANOVA with Tukey's multiple comparisons test. *p<0.05, **p<0.01, ***p<0.001, ****p<0.0001.*

*B) Maximum force generated by pumping fluid at various flow rates through the model. N= 3; data shown is mean ± standard deviation. Ordinary one-way ANOVA with Tukey's multiple comparisons test. *p<0.05, **p<0.01, ***p<0.001, ****p<0.0001.*

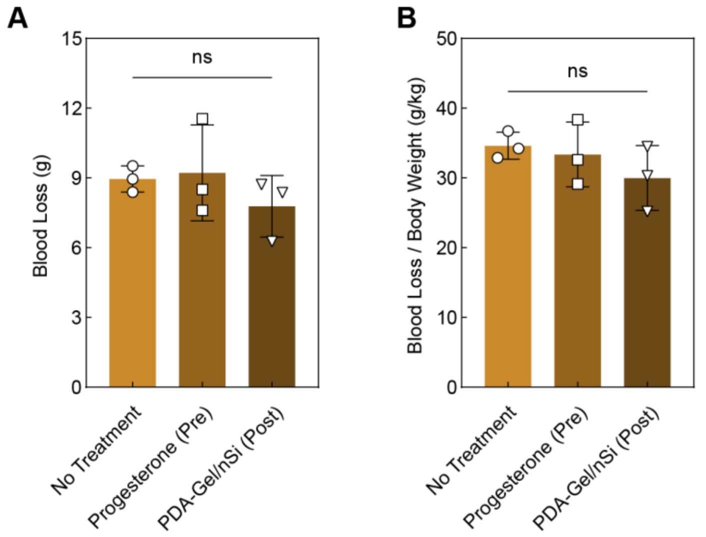

Figure S15. Blood loss measurements for rat uterine laceration model.

*A) Raw blood loss measurements for rats in no treatment, progesterone pre-treatment, and PDA-gel/nSi hydrogel treatment groups. N = 3; data shown is mean ± standard deviation. Ordinary one-way ANOVA with Tukey's multiple comparisons test. *p<0.05, **p<0.01, ***p<0.001, ****p<0.0001.*

*B) Blood loss normalized to rat body weight shown in grams of blood loss per kilogram of body weight. Note that data is from the same data as shown in Figure S15A. N = 3; data shown is mean ± standard deviation. Ordinary one-way ANOVA with Tukey's multiple comparisons test. *p<0.05, **p<0.01, ***p<0.001, ****p<0.0001.*

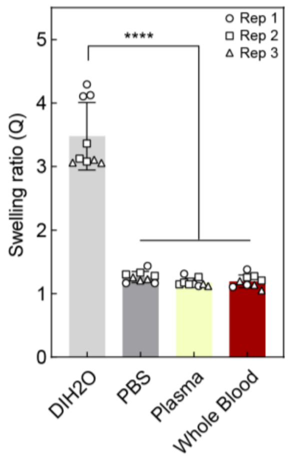

Figure S16. Swelling ratio of PDA-gel/nSi hydrogel in physiological solutions.

*Swelling ratio of the PDA-gel/nSi hydrogel is shown in deionized water (DIH2O), PBS, human plasma, and whole human blood. Citrated whole blood was used without recalcification in order to isolate the swelling effect without mass increases due to clot formation. N = 9 across three unique iterations (N = 3 samples per replicate experiment; unique blood donor selected for each replicate experiment); data shown is mean ± standard deviation. Ordinary one-way ANOVA with Tukey's multiple comparisons test. *p<0.05, **p<0.01, ***p<0.001, ****p<0.0001.*

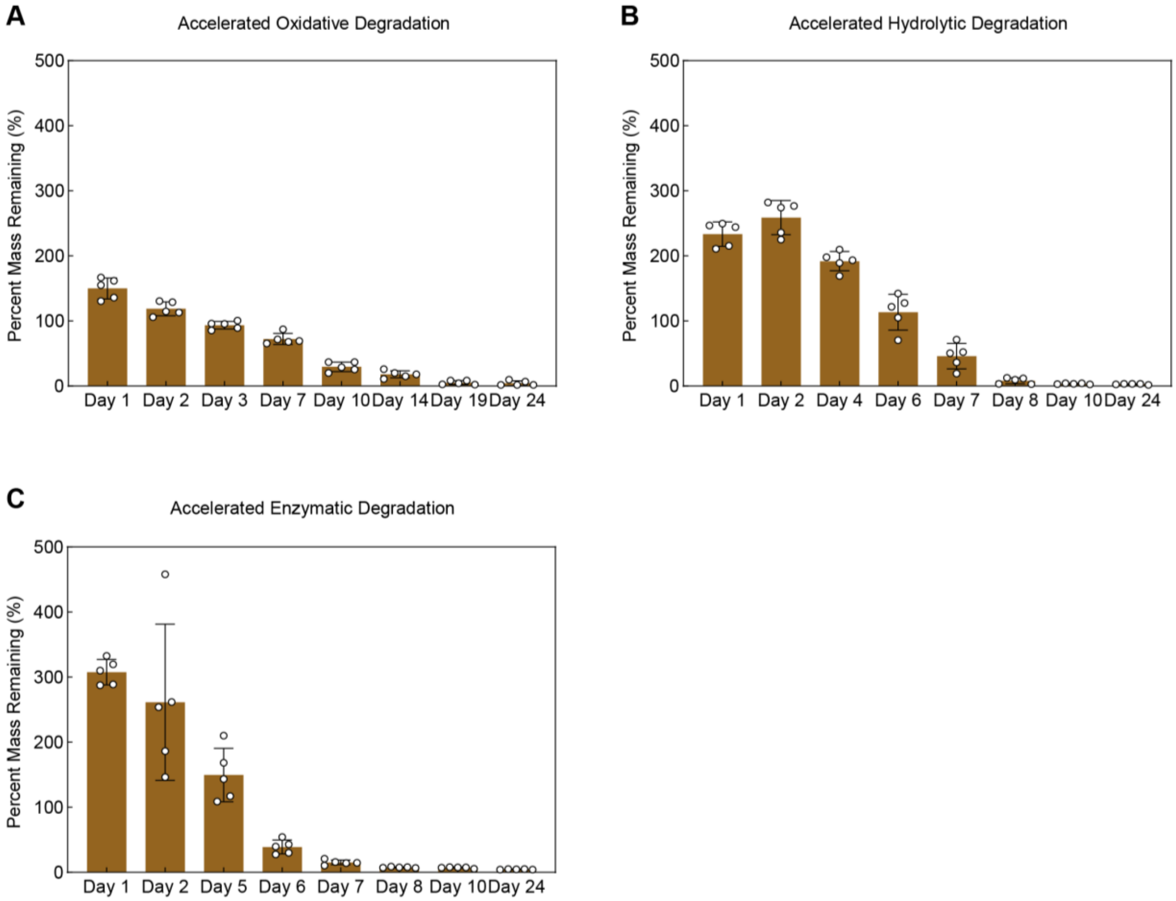

Figure S17. Accelerated degradation studies of PDA-gel/nSi hydrogels.

A) Accelerated oxidative degradation conducted in 20% H_2O_2. Timepoints are selected to highlight swelling during first two days and steady mass loss thereafter. N = 5; data shown is mean ± standard deviation.

B) Accelerated hydrolytic degradation conducted in 0.1 M NaOH. Timepoint are selected to highlight swelling over first six days followed by rapid mass loss thereafter. N = 5; data shown is mean ± standard deviation.

C) Accelerated enzymatic degradation conducted in 5 U/mL collagenase. Timepoints are selected to highlight swelling over first five days followed by rapid mass loss thereafter. N = 5; data shown is mean ± standard deviation.

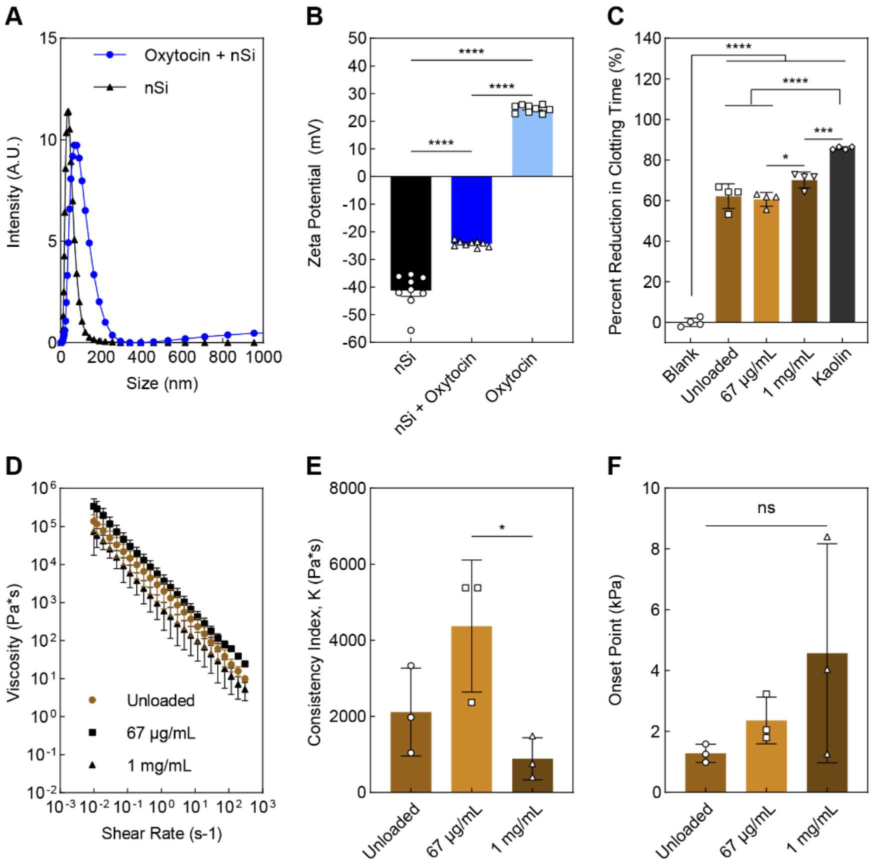

Figure S18. Hydrogel augmentation with oxytocin.

A) Dynamic light scattering of nanosilicate (black triangles) and nanosilicate with oxytocin (blue circles), showing an increase in particle size with the addition of oxytocin. N = 3 material replicates with three measurements per replicate; data shown is mean. Standard error is not shown for visual clarity but statistical analysis is available in Zenodo repository.

*B) Zeta potential of nanosilicate, oxytocin, and nanosilicate with oxytocin, showing a change in zeta potential with the complexation of oxytocin and nanosilicate. N = 3 material replicates with three measurements per replicate; data shown is mean ± standard error of the mean. Ordinary one-way ANOVA with Tukey's multiple comparisons test. *p<0.05, **p<0.01, ***p<0.001, ****p<0.0001.*

*C) Bovine blood clotting time inversion test for unloaded PDA-Gel/nSi hydrogel, PDA-Gel/nSi hydrogel loaded with 67 μg/mL oxytocin, PDA-Gel/nSi hydrogel loaded with 1 mg/mL oxytocin. Blank tubes served as a negative control; kaolin served as a positive control. Data is represented as percent reduction from blank tubes. N = 4; data shown is mean ± standard deviation. Ordinary one-way ANOVA with Tukey's multiple comparisons test. *p<0.05, **p<0.01, ***p<0.001, ****p<0.0001.*

D) Shear rate sweep of PDA-Gel/nSi hydrogel, PDA-Gel/nSi hydrogel loaded with 67 μg/mL oxytocin, PDA-Gel/nSi hydrogel loaded with 1 mg/mL oxytocin at 37°C. N = 3; data shown is mean ± standard deviation. Note that data for the unloaded hydrogel is duplicated from Figure 1G.

*E) Consistency index, K, derived from a power-law fit to the viscosity-vs-shear rate curves in Figure S21D, indicating the theoretical viscosity of an equivalent Newtonian fluid. N = 3; data shown is mean ± standard deviation. Ordinary one-way ANOVA with Tukey's multiple comparisons test. *p<0.05, **p<0.01, ***p<0.001, ****p<0.0001. Note that data for the unloaded hydrogel is duplicated from Figure S6.*

F) Onset point of storage modulus from stress sweeps at 37°C for PDA-Gel/nSi hydrogel, PDA-Gel/nSi hydrogel loaded with 67 μg/mL oxytocin, PDA-Gel/nSi hydrogel loaded with 1 mg/mL oxytocin. N = 3; data shown is mean ± standard deviation. Note that data for the unloaded hydrogel is duplicated from Figure S10.

Supporting Information Video 1. No treatment uterine laceration.

Video of uterine laceration surgery for no treatment group. Video is displayed at 20X speed. Contractions are visible in the soft uterine tissue.

Supporting Information Video 2. Progesterone pre-treatment uterine laceration.

Video of uterine laceration surgery for progesterone pre-treatment group. Video is displayed at 20X speed. Contractions are visible in the soft uterine tissue.

Supporting Information Video 3. PDA-gel/nSi hydrogel treatment uterine laceration.

Video of uterine laceration surgery for PDA-gel/nSi hydrogel treatment group. Video is displayed at 20X speed. Contractions are visible in the soft uterine tissue. The hydrogel is noted to remain adhered to the tissue throughout this movement.

REFERENCES

(1) Gowda, A. H. J.; Bu, Y.; Kudina, O.; Krishna, K. V.; Bohara, R. A.; Eglin, D.; Pandit, A. Design of tunable gelatin-dopamine based bioadhesives. *Int J Biol Macromol* **2020**, *164*, 1384–1391. DOI: 10.1016/j.ijbiomac.2020.07.195 From NLM Medline.

(2) Fan, C.; Wang, D.-A. Novel Gelatin-based Nano-gels with Coordination-induced Drug Loading for Intracellular Delivery. *Journal of Materials Science & Technology* **2016**, *32* (9), 840–844. DOI: 10.1016/j.jmst.2016.04.009.

(3) Fan, C.; Fu, J.; Zhu, W.; Wang, D. A. A mussel-inspired double-crosslinked tissue adhesive intended for internal medical use. *Acta Biomater* **2016**, *33*, 51–63. DOI: 10.1016/j.actbio.2016.02.003 From NLM Medline.

(4) Yang, X.; Zhu, L.; Tada, S.; Zhou, D.; Kitajima, T.; Isoshima, T.; Yoshida, Y.; Nakamura, M.; Yan, W.; Ito, Y. Mussel-inspired human gelatin nanocoating for creating biologically adhesive surfaces. *Int J Nanomedicine* **2014**, *9*, 2753–2765. DOI: 10.2147/IJN.S60624 From NLM Medline.

(5) Fu, J.; Quek, K. Y.; Chuah, Y. J.; Lim, C. S.; Fan, C.; Wang, D. A. The effects of gelatin-dopamine coating on polydimethylsiloxane substrates on pluripotency maintenance and myocardial differentiation of cultured mouse embryonic stem cells. *J Mater Chem B* **2016**, *4* (48), 7961–7973. DOI: 10.1039/c6tb02631a From NLM PubMed-not-MEDLINE.

(6) Liu, Y.; Cheong Ng, S.; Yu, J.; Tsai, W. B. Modification and crosslinking of gelatin-based biomaterials as tissue adhesives. *Colloids Surf B Biointerfaces* **2019**, *174*, 316–323. DOI: 10.1016/j.colsurfb.2018.10.077 From NLM Medline.

(7) Jin, A.; Wang, Y.; Lin, K.; Jiang, L. Nanoparticles modified by polydopamine: Working as "drug" carriers. *Bioact Mater* **2020**, *5* (3), 522–541. DOI: 10.1016/j.bioactmat.2020.04.003 From NLM PubMed-not-MEDLINE.

(8) Zhang, K.; Wei, Z.; Xu, X.; Feng, Q.; Xu, J.; Bian, L. Efficient catechol functionalization of biopolymeric hydrogels for effective multiscale bioadhesion. *Mater Sci Eng C Mater Biol Appl* **2019**, *103*, 109835. DOI: 10.1016/j.msec.2019.109835 From NLM Medline.

(9) Arnow, L. E. Colorimetric Determination of the Components of 3,4-Dihydroxyphenylalaninetyrosine Mixtures. *Journal of Biological Chemistry* **1937**, *118* (2), 531–537. DOI: 10.1016/s0021-9258(18)74509-2.

(10) *Carbodiimide Crosslinker Chemistry*. Thermo Fisher Scientific, 2026. https://www.thermofisher.com/us/en/home/life-science/protein-biology/protein-biology-learning-center/protein-biology-resource-library/pierce-protein-methods/carbodiimide-crosslinker-chemistry.html (accessed 2026 January 5, 2026).

(11) Wu, M.; Wang, T.; Müller, L.; Müller, F. A. Adjustable synthesis of polydopamine nanospheres and their nucleation and growth. *Colloids and Surfaces A: Physicochemical and Engineering Aspects* **2020**, *603*. DOI: 10.1016/j.colsurfa.2020.125196.

(12) de Barros, N. R.; Gangrade, A.; Elsebahy, A.; Chen, R.; Zehtabi, F.; Ermis, M.; Falcone, N.; Haghniaz, R.; Khosravi, S.; Gomez, A.; et al. Injectable Nanoengineered Adhesive Hydrogel for Treating Enterocutaneous Fistulas. *Acta Biomater* **2024**, *173*, 231–246. DOI: 10.1016/j.actbio.2023.10.026 From NLM Medline.

(13) Liu, L.; Deng, D.; Xing, Y.; Li, S.; Yuan, B.; Chen, J.; Xia, N. Activity analysis of the carbodiimide-mediated amine coupling reaction on self-assembled monolayers by cyclic voltammetry. *Electrochimica Acta* **2013**, *89*, 616–622. DOI: 10.1016/j.electacta.2012.11.049.

(14) Montazerian, H.; Baidya, A.; Haghniaz, R.; Davoodi, E.; Ahadian, S.; Annabi, N.; Khademhosseini, A.; Weiss, P. S. Stretchable and Bioadhesive Gelatin Methacryloyl-Based Hydrogels Enabled by in Situ Dopamine Polymerization. *ACS Appl Mater Interfaces* **2021**, *13* (34), 40290–40301. DOI: 10.1021/acsami.1c10048 From NLM Medline.

(15) Lim, J.; Zhang, S.; Heo, J. M.; Dickwella Widanage, M. C.; Ramamoorthy, A.; Kim, J. Polydopamine Adhesion: Catechol, Amine, Dihydroxyindole, and Aggregation Dynamics. *ACS Appl Mater Interfaces* **2024**, *16* (24), 31864–31872. DOI: 10.1021/acsami.4c08603 From NLM Medline.

(16) Braunstein, K. M.; Sarji, K. E.; Kleinfelder, J.; Scraibman, H. B.; Colwell, J. A.; Eurenius, K. The effects of dopamine on human platelet aggregation, in vitro. *J Pharmacol Exp Ther* **1977**, *200* (2), 449–457.
(17) Cao, S.; Li, Q.; Zhang, S.; Liu, Z.; Lv, X.; Chen, J. Preparation of biodegradable carboxymethyl cellulose/dopamine/Ag NPs cryogel for rapid hemostasis and bacteria-infected wound repair. *Int J Biol Macromol* **2022**, *222* (Pt A), 272–284. DOI: 10.1016/j.ijbiomac.2022.09.172 From NLM Medline.
(18) Hargett, S. E.; Lokhande, G. K.; Duran, J.; Hirani, Z.; Jang, L. K.; Foster, S.; Deo, K. A.; George, S.; Javed, M.; Ware, T. H.; et al. Nanoengineered Shape-Memory Hemostat. *Small Sci* **2025**, *5* (2), 2400321. DOI: 10.1002/smsc.202400321 From NLM PubMed-not-MEDLINE.
(19) Biswas, S.; Miller, S. E.; Roy, S.; Thazhaiselvam, J.; Foster, S.; Kalairaj, M. S.; George, S. M.; Jones-Hall, Y.; Horn, S. J.; Clubb, F. J.; et al. Expandable Nanocomposite Shape-Memory Hemostat for the Treatment of Noncompressible Hemorrhage. *Adv Sci (Weinh)* **2026**, e08439. DOI: 10.1002/advs.202508439 From NLM Publisher.
(20) Avery, R. K.; Albadawi, H.; Akbari, M.; Zhang, Y. S.; Duggan, M. J.; Sahani, D. V.; Olsen, B. D.; Khademhosseini, A.; Oklu, R. An injectable shear-thinning biomaterial for endovascular embolization. *Science Translational Medicine* **2016**, *8* (365). DOI: 10.1126/scitranslmed.aah5533.
